# Application of whole genome data for in silico evaluation of primers and probes routinely employed for the detection of viral species by RT-qPCR using dengue virus as a case study

**DOI:** 10.1186/s12859-018-2313-0

**Published:** 2018-09-04

**Authors:** Kevin Vanneste, Linda Garlant, Sylvia Broeders, Steven Van Gucht, Nancy H. Roosens

**Affiliations:** 1 Transversal activities in applied genomics, Sciensano, (1050) Brussels, Belgium; 2 Viral Diseases, Sciensano, (1050) Brussels, Belgium; 3 Present address: Quality of Laboratories, Sciensano, (1050) Brussels, Belgium

**Keywords:** Dengue virus, RT-qPCR, BLAST, Virus detection

## Abstract

**Background:**

Viral infection by dengue virus is a major public health problem in tropical countries. Early diagnosis and detection are increasingly based on quantitative reverse transcriptase real-time polymerase chain reaction (RT-qPCR) directed against genomic regions conserved between different isolates. Genetic variation can however result in mismatches of primers and probes with their targeted nucleic acid regions. Whole genome sequencing allows to characterize and track such changes, which in turn enables to evaluate, optimize, and (re-)design novel and existing RT-qPCR methods. The immense amount of available sequence data renders this however a labour-intensive and complex task.

**Results:**

We present a bioinformatics approach that enables in silico evaluation of primers and probes intended for routinely employed RT-qPCR methods. This approach is based on analysing large amounts of publically available whole genome data, by first employing BLASTN to mine the genomic regions targeted by the RT-qPCR method(s), and afterwards using BLASTN-SHORT to evaluate whether primers and probes will anneal based on a set of simple in silico criteria. Using dengue virus as a case study, we evaluated 18 published RT-qPCR methods using more than 3000 publically available genomes in the NCBI Virus Variation Resource, and provide a systematic overview of method performance based on in silico sensitivity and specificity.

**Conclusions:**

We provide a comprehensive overview of dengue virus RT-qPCR method performance that will aid appropriate method selection allowing to take specific measures that aim to contain and prevent viral spread in afflicted regions. Notably, we find that primer-template mismatches at their 3′ end may represent a general issue for dengue virus RT-qPCR detection methods that merits more attention in their development process. Our approach is also available as a public tool, and demonstrates how utilizing genomic data can provide meaningful insights in an applied public health setting such as the detection of viral species in human diagnostics.

**Electronic supplementary material:**

The online version of this article (10.1186/s12859-018-2313-0) contains supplementary material, which is available to authorized users.

## Background

Dengue virus is a mosquito-borne single positive-stranded RNA flavivirus comprising five distinct serotypes [1], all of which cause a spectrum of diseases [2] ranging from a mild, self-limiting febrile illness (dengue fever) to more severe forms characterized by a high mortality rate (dengue haemorrhagic fever and shock syndrome) [3]. As the viremia lasts only 3 days after initial infection, early detection is crucial to diagnose the disease, apply appropriate treatment and take necessary vector-control measures [4]. Symptoms of dengue fever are however mostly aspecific, and reliable diagnosis is difficult because techniques based on immunological assays are plagued by possible cross-reaction of antibodies with other members of the *Flavivirus* genus [5].

Among diagnostic tests for early discovery, RNA detection by quantitative reverse transcriptase real-time polymerase chain reaction (RT-qPCR) represents a fast, specific and sensitive tool for the management of acute infections, surveillance and outbreak investigations allowing both detection and quantification of viral RNA [6]. The appropriate mix of specifically designed primers and probes can even allow to differentiate between different serotypes by using a unique multiplex reaction [7]. Flaviviruses can however adapt quickly to selective pressures through error-prone replication introducing nucleotide substitutions that modulate genetic variation within the population [8]. Developed RT-qPCR methods must therefore be validated in the laboratory on a large set of reference samples to verify that the targeted genomic regions are adequately conserved within the species or serotype depending on the desired resolution. Traditionally, a limited number of reference samples were however used in the experimental validation of routinely employed (RT-)qPCR methods (e.g. [9]), which is unlikely to represent the entire pool of standing genetic variation [10].

Next-Generation Sequencing (NGS), also referred to as High-Throughput Sequencing (HTS), has become a widely available technology with reduced costs and higher throughput compared to conventional Sanger sequencing, providing an increasing number of Whole Genome Sequencing (WGS) data [11]. This allows to track genomic changes with a resolution up to the single nucleotide so that variation within a viral population can now be determined with digital precision. For dengue virus, more than 3000 whole genomes are currently publically available in the NCBI Virus Variation Resource [12], providing a valuable resource documenting (part of) the standing genetic variation within the population. This allows systematic re-evaluation of previously developed RT-qPCR methods that are routinely employed in order to investigate their feasibility for capturing their intended genomic targets in face of the currently known genetic variation, and enhancing previously and newly developed methods through a cyclic process of optimization based on employing WGS data.

Such systematic investigations however present a substantial bottleneck for routine enforcement laboratories, which often do not have access to the required bioinformatics expertise and/or resources, especially when considering the intricacies encountered in the proper design of primers and probes [13]. Manual alignment of primers and probes to thousands of individual genomes would require an enormous time investment. Many tools have already been developed for assisting the process of primer and probe design and evaluation. Primer3 is for instance a well-known program for designing primers based on a variety of parameters and options [14]. Other popular tools such as In-Silico PCR (available at https://genome.ucsc.edu/cgi-bin/hgPcr), Primer-Blast [15], and FastPCR [16], allow to simulate the PCR process in silico, enabling to investigate the amplification targets of primers and probes to ensure adequate sensitivity and specificity. Many other tools exist (see https://omictools.com/qpcr-category for an up-to-date overview). Although these tools provide previously unrivalled possibilities for designing and evaluating primers and probes, they are typically not tailored towards the need for quickly analysing multiple genomes to assess whether a RT-qPCR method will lead to a signal or not in each individual genome. This renders systematic evaluation of different RT-qPCR methods still a labour-intensive task that is out of scope for most routine enforcement specialists working with such methods in daily practise.

We present an approach that enables in silico evaluation of primers and probes intended for routinely employed RT-qPCR methods by utilizing publically available whole genome data. This method first extracts the targeted genomic regions in the analysed genomes and then assesses whether primers and probes will successfully anneal resulting in signal detection. We evaluated 18 published RT-qPCR methods for dengue virus detection, employing more than 3000 genomes, and provide the first systematic overview of RT-qPCR method performance for this viral species. This approach will aid the development of methods better suited for the detection of viruses in human diagnostics, as well as other fields that rely on (RT-q)PCR.

## Results

**Fig. 1:**
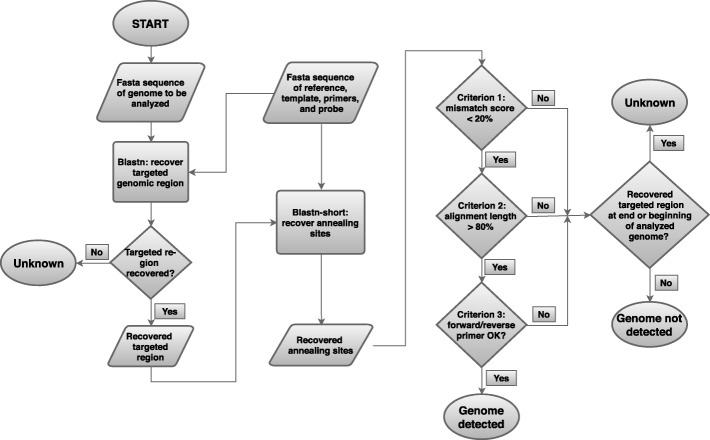
Overview of the workflow for in silico evaluation of RT-qPCR methods. A two-step BLAST approach is used to first recover the genomic regions targeted by the RT-qPCR method under investigation in every analysed genome, after which the annealing regions for the primers and probe are extracted. Hybridisation properties of the primer/probe-template pair are then investigated by means of a set of selection criteria that mimic the PCR reaction: mismatch percentage (a maximum of 20% of bases can be mismatched in the primer/probe), alignment length (a minimum primer/probe alignment length of 80% is required), and number of mismatched bases in the 3′ end region of primers (either one or no single mismatch is allowed in the last five bases of this region). Threshold values for these selection criteria were set in accordance with previous observations documented in the literature (see Discussion). Genomes are considered as detected only if all three criteria are met, and are otherwise classified as not detected. Unknown cases represent genomes where the targeted genomic region cannot be extracted, because it either is not present or alternatively incomplete and located at the beginning or end of the genomic sequence. See Methods for an extended description of the workflow

A literature review was performed to collect information for 18 RT-qPCR methods for dengue virus detection (see Table 1), while whole genome sequences were collected directly from the NCBI Virus Variation Resource [12]. Method performance was assessed based on an in silico workflow (see Fig. 1) that was applied on all available complete genomes for every individual RT-qPCR method. This workflow uses a two-step BLAST approach by first retrieving the targeted genomic region based on a template reference sequence, and afterwards extracting the annealing sites within the recovered region based on the sequences of the primers and probe (see Methods). Three criteria evaluate afterwards whether the primers and probe of the RT-qPCR method will anneal and result in (theoretical) detection. First, the number of mismatches in the annealing sites of primers and probe should be lower than 20% (relative to the total length of the primer or probe). Second, the length of the annealing sites for primers and probe should constitute at least 80% of their total length. These two criteria were introduced to account for the observation that the PCR reaction is relatively robust to primer/probe-template mismatches, but that an increasing number of mismatched bases will progressively adversely affect the PCR reaction (see also Discussion) [17, 18]. Third, both the forward and reverse primers should not contain more than a predefined number of mismatches in the last five bases at their 3′ end. This criterion was introduced to account for the observation that mismatch tolerance in primer-template pairs is much lower towards their 3′ end. Because two mismatches at the 3’ end generally prevent amplification [10, 19], the workflow was run twice for all RT-qPCR methods while allowing either no or one such mismatch (see also Discussion). Only if all three criteria are passed successfully, the genome under investigation is considered as being detected by the RT-qPCR method. Otherwise, the genome is considered as either not detected or unknown. The former category represents cases where one or more criteria are not passed. The latter category represents genomes where no targeted genomic region could be recovered, or alternatively such a region was recovered but located at either the beginning or end of the genomic sequence. Unknown cases can be due to the genome not containing a region compatible with the RT-qPCR method, or alternatively the genomic sequence itself being incomplete. Discrimination between both fates is impossible without detailed investigation within the laboratory, but the strong and statistically significant overrepresentation of unknown cases for RT-qPCR methods that target genomic regions located at either the beginning or end of genomes, does strongly suggest that this is caused by the genomic sequences being incomplete rather than the targeted genomic regions not being present (see Additional file 1). RT-qPCR method performance was then scored by means of in silico sensitivity and specificity.

**Table 1:** List of evaluated RT-qPCR methods for dengue virus detection

Method name	Targeted genomic region	Reference publication
Callahan_1_g	3’UTR	[31]
Callahan_2_g	3’UTR	
Callahan_3_g	3’UTR	
Callahan_4_g	3’UTR	
Callahan_1_s	NS5	[31]
Callahan_2_s	capsid	
Callahan_3_s	capsid	
Callahan_4_s	capsid	
Cecilia_4_s	3’ UTR	[25]
Chien_1_s	NS5	[26]
Chien_2_s	NS5	
Chien_3_s	NS5	
Chien_4_s	NS5	
Conceicao_1_g	5’UTR	[20]
Conceicao_2_g	5’UTR	
Conceicao_3_g	5’UTR	
Conceicao_4_g	5’UTR	
Drosten_1_g	3’ UTR	[21]
Drosten_2_g	3’ UTR	
Drosten_3_g	3’ UTR	
Drosten_4_g	3’ UTR	
Gurukumar_1_g	3’ UTR	[22]
Gurukumar_2_g	3’ UTR	
Gurukumar_3_g	3’ UTR	
Gurukumar_4_g	3’ UTR	
Ito_1_s	E	[27]
Ito_2_s	E	
Ito_3_s	E	
Ito_4_s	E	
Johnson_1_s	N5S	[7]
Johnson_2_s	E	
Johnson_3_s	prM	
Johnson_4_s	prM	
Kim_1_s	NS1	[33]
Kim_2_s	NS1	
Kim_3_s	NS1	
Kim_4_s	NS1	
Kong_1_s	NS5	[28]
Kong_2_s	NS5	
Kong_3_s	NS5	
Kong_4_s	NS5	
Laue_1_s	3’ UTR	[34]
Laue_2_s	3’ UTR	
Laue_3_s	3’ UTR	
Laue_4_s	3’ UTR	
Leparc_Goffart_1_g	3’UTR	[32]
Leparc_Goffart_2_g	3’UTR	
Leparc_Goffart_3_g	3’UTR	
Leparc_Goffart_4_g	3’UTR	
Leparc_Goffart_1_s	capsid	[32]
Leparc_Goffart_2_s	capsid	
Leparc_Goffart_3_s	capsid	
Leparc_Goffart_4_s	capsid	
Pongsiri_1_g	3’UTR	[23]
Pongsiri_2_g	3’UTR	
Pongsiri_3_g	3’UTR	
Pongsiri_4_g	3’UTR	
Sadon_1_s	capsid	[29]
Sadon_2_s	capsid	
Sadon_3_s	capsid	
Sadon_4_s	capsid	
Santiago_1_s	NS5	[30]
Santiago_2_s	E	
Santiago_3_s	prM	
Santiago_4_s	prM	
Warrilow_1_g	3’UTR	[24]
Warrilow_2_g	3’UTR	
Warrilow_3_g	3’UTR	
Warrilow_4_g	3’UTR	

The first column lists the adapted method name. Methods for dengue virus and serotype-specific detection are labelled with the extensions ‘_g’ and ‘_s’, respectively. Each method is subdivided in the four dengue virus serotypes to evaluate each serotype individually, even for methods designed to detect the entire species. The method ‘Cecilia_4_s’ was only evaluated for the fourth serotype as it was specifically designed towards this purpose [25]. The second column lists the targeted genomic region. The third column lists the reference publication for each method. See also Additional file 1: Table S1 for detailed sequence information for primers, probe and reference template, for every method

For the in silico sensitivity, all 18 RT-qPCR methods were challenged with their target genomes. A more and a less conservative score were obtained for every RT-qPCR method by either including unknown cases as genomes not being detected, or excluding them from the analysis (see Methods). Table 2 presents results when allowing for either one or no single mismatch in the last five bases of primer annealing sites at their 3′ end (all other thresholds were kept constant). Table 2 demonstrates that when using the more and the less conservative score, between nine and 16 out of 18 methods, respectively, exhibit an average in silico sensitivity > 95% when one mismatch in the last five bases at the 3′ end was allowed. Based on the less conservative score, this applies for the methods developed by Conceicao et al. [20], Drosten et al. [21], Gurukumar et al. [22], Pongsiri et al. [23], and Warrilow et al. [24] for dengue virus detection; Cecilia et al. [25], Chien et al. [26], Ito et al. [27], Johnson et al. [7], Kong et al. [28], Sadon et al. [29], and Santiago et al. [30] for serotype-specific detection; and Callahan et al. [31] and Leparc-Goffart et al. [32] for both dengue virus and serotype-specific detection. The two remaining methods not meeting this threshold of 95% in silico sensitivity based on the less conservative score are the methods developed by Kim et al. [33] and Laue et al. [34] for serotype-specific detection. Table 2 also illustrates that when no mismatches in the last five bases of primer annealing sites at the 3′ end were allowed, using the more and the less conservative score, only between three and eight out of 18 methods, respectively, exhibit an average in silico sensitivity > 95%. Based on the less conservative score, this applies for the methods developed by Callahan et al. [31], Conceicao et al. [20], Drosten et al. [21], Pongsiri et al. [23], and Warrilow et al. [24] for dengue virus detection; Santiago et al. [30] for serotype-specific detection; and Leparc-Goffart et al. [32] for both dengue virus and serotype-specific detection. The 10 remaining methods not meeting this threshold of 95% in silico sensitivity based on the less conservative score are the methods developed by Gurukumar et al. [22] for dengue virus detection; and Callahan et al. [31], Cecilia et al. [25], Chien et al. [26], Ito et al. [27], Johnson et al. [7], Kim et al. [33], Kong et al. [28], Laue et al. [34], and Sadon et al. [29] for serotype-specific detection. The method developed by Chien et al. [26] for serotype-specific detection performs particularly poorly with a more and a less conservative score of both 6.95%, whereas this was 99.11% when one mismatch in the last five bases at the 3′ end was allowed. This indicates a marked effect of this criterion on method performance. Further inspection revealed that this difference is caused by the forward primer, reverse primer, and both primers, exhibiting nucleotide mismatches within the last five nucleotides at their 3′ end for serotype 3, serotype 1, and serotype 2, respectively (see Additional file 1). Notably, this method scores well for detection of the fourth serotype. Other methods for both dengue virus or serotype-specific detection with a low average in silico sensitivity display a similar trend by scoring well on particular serotypes but poorly on others, frequently due to mismatches at the 3′ end of primer-template pairs.

**Table 2:** Dengue virus RT-qPCR method performance in terms of in silico sensitivity

Method name	# genomes analysed	One mismatch allowed at 3′ end of primer-template pairs					No mismatches allowed at 3′ end of primer-template pairs				
		# genomes detected	# genomes not detected	# genomes unknown	% in silico sensitivity	% average in silico sensitivity	# genomes detected	# genomes not detected	# genomes unknown	% in silico sensitivity	% average in silico sensitivity
Callahan_1_g	1359	1209	14	136	[88.96–98.86]	[90.12–98.80]	1206	17	136	[88.74–98.61]	[89.80–98.46]
Callahan_2_g	1164	1055	13	96	[90.64–98.78]		1054	14	96	[90.55–98.69]	
Callahan_3_g	777	707	11	59	[90.99–98.47]		704	14	59	[90.60–98.05]	
Callahan_4_g	182	167	0	15	[91.76–100]		163	4	15	[89.56–97.60]	
Callahan_1_s	1359	1356	3	0	[99.78–99.78]	[99.48–99.48]	1287	72	0	[94.70–94.70]	[81.99–81.99]
Callahan_2_s	1164	1150	14	0	[98.80–98.80]		638	526	0	[54.81–54.81]	
Callahan_3_s	777	777	0	0	[100–100]		777	0	0	[100–100]	
Callahan_4_s	182	181	1	0	[99.45–99.45]		153	29	0	[84.07–84.07]	
Cecilia_4_s	182	153	4	25	[84.07–97.45]	[84.07–97.45]	121	36	25	[66.48–77.07]	[66.48–77.07]
Chien_1_s	1359	1357	2	0	[99.85–99.85]	[99.11–99.11]	4	1355	0	[0.29–0.29]	[6.95–6.95]
Chien_2_s	1164	1138	26	0	[97.77–97.77]		1	1163	0	[0.09–0.09]	
Chien_3_s	777	777	0	0	[100–100]		58	719	0	[7.46–7.46]	
Chien_4_s	182	179	3	0	[98.35–98.35]		179	3	0	[98.35–98.35]	
Conceicao_1_g	1359	1359	0	0	[100–100]	[99.91–99.91]	1354	5	0	[99.63–99.63]	[95.23–95.23]
Conceicao_2_g	1164	1163	1	0	[99.91–99.91]		1159	5	0	[99.57–99.57]	
Conceicao_3_g	777	776	1	0	[99.87–99.87]		623	154	0	[80.18–80.18]	
Conceicao_4_g	182	181	1	0	[99.45–99.45]		180	2	0	[98.90–98.90]	
Drosten_1_g	1359	1088	48	223	[80.06–95.77]	[83.77–98.22]	1082	51	226	[79.62–95.50]	[82.60–97.26]
Drosten_2_g	1164	1012	3	149	[86.94–99.70]		979	28	157	[84.11–97.22]	
Drosten_3_g	777	660	1	116	[84.94–99.85]		658	1	118	[84.68–99.85]	
Drosten_4_g	182	157	1	24	[86.26–99.37]		157	1	24	[86.26–99.37]	
Gurukumar_1_g	1359	1082	48	229	[79.62–95.75]	[79.64–96.35]	1074	53	232	[79.03–95.30]	[77.51–94.27]
Gurukumar_2_g	1164	1003	4	157	[86.17–99.60]		969	31	164	[83.25–96.90]	
Gurukumar_3_g	777	655	1	121	[84.30–99.85]		650	1	126	[83.66–99.85]	
Gurukumar_4_g	182	33	52	97	[18.13–38.82]		6	79	97	[3.30–7.06]	
Ito_1_s	1359	1353	6	0	[99.56–99.56]	[99.43–99.43]	0	1359	0	[0.00–0.00]	[57.18–57.18]
Ito_2_s	1164	1150	14	0	[98.80–98.80]		1075	89	0	[92.35–92.35]	
Ito_3_s	777	777	0	0	[100–100]		737	40	0	[94.85–94.85]	
Ito_4_s	182	182	0	0	[100–100]		179	3	0	[98.35–98.35]	
Johnson_1_s	1359	1357	2	0	[99.85–99.85]	[98.56–98.59]	1125	234	0	[82.78–82.78]	[69.04–69.06]
Johnson_2_s	1164	1150	13	1	[98.80–98.88]		1125	38	1	[96.65–96.73]	
Johnson_3_s	777	748	29	0	[96.27–96.27]		10	767	0	[1.29–1.29]	
Johnson_4_s	182	177	5	0	[97.25–97.25]		144	38	0	[79.12–79.12]	
Kim_1_s	1359	914	445	0	[67.26–67.26]	[86.53–86.53]	528	831	0	[38.85–38.85]	[60.71–60.71]
Kim_2_s	1164	1146	18	0	[98.45–98.45]		1094	70	0	[93.99–93.99]	
Kim_3_s	777	774	3	0	[99.61–99.61]		327	450	0	[42.08–42.08]	
Kim_4_s	182	179	3	0	[98.35–98.35]		165	17	0	[90.66–90.66]	
Kong_1_s	1359	1254	105	0	[92.27–92.27]	[96.12–96.12]	0	1359	0	[0.00–0.00]	[28.12–28.12]
Kong_2_s	1164	1147	17	0	[98.54–98.54]		832	332	0	[71.48–71.48]	
Kong_3_s	777	775	2	0	[99.74–99.74]		0	777	0	[0.00–0.00]	
Kong_4_s	182	171	11	0	[93.96–93.96]		147	35	0	[80.77–80.77]	
Laue_1_s	1359	1358	1	0	[99.93–99.93]	[86.56–86.56]	1027	332	0	[75.57–75.57]	[69.30–69.30]
Laue_2_s	1164	1149	15	0	[98.71–98.71]		1000	164	0	[85.91–85.91]	
Laue_3_s	777	327	450	0	[42.08–42.08]		232	545	0	[29.86–29.86]	
Laue_4_s	182	180	2	0	[98.90–98.90]		154	28	0	[84.62–84.62]	
Leparc_Goffart_1_g	1359	1231	28	100	[90.58–97.78]	[91.07–98.36]	1225	33	101	[90.14–97.38]	[90.67–97.95]
Leparc_Goffart_2_g	1164	1058	14	92	[90.89–98.69]		1057	15	92	[90.81–98.60]	
Leparc_Goffart_3_g	777	715	11	51	[92.02–98.48]		712	14	51	[91.63–98.07]	
Leparc_Goffart_4_g	182	167	0	15	[91.76–100]		163	4	15	[89.56–97.60]	
Leparc_Goffart_1_s	1359	1358	1	0	[99.93–99.93]	[80.18–99.93]	1340	19	0	[98.60–98.60]	[77.83–96.99]
Leparc_Goffart_2_s	1164	475	1	688	[40.81–99.79]		446	30	688	[38.32–93.70]	
Leparc_Goffart_3_s	777	777	0	0	[100–100]		775	2	0	[99.74–99.74]	
Leparc_Goffart_4_s	182	182	0	0	[100–100]		149	33	0	[81.87–81.87]	
Pongsiri_1_g	1359	1291	12	56	[95.00–99.08]	[95.98–99.32]	1287	16	56	[94.70–98.77]	[95.81–99.17]
Pongsiri_2_g	1164	1121	9	34	[96.31–99.20]		1122	8	34	[96.39–99.29]	
Pongsiri_3_g	777	750	2	25	[96.53–99.73]		748	4	25	[96.27–99.47]	
Pongsiri_4_g	182	180	0	2	[98.90–100]		179	0	3	[98.35–100]	
Sadon_1_s	1359	1358	1	0	[99.93–99.93]	[99.66–99.66]	1251	108	0	[92.05–92.05]	[88.91–88.91]
Sadon_2_s	1164	1158	6	0	[99.48–99.48]		1004	160	0	[86.25–86.25]	
Sadon_3_s	777	777	0	0	[100–100]		666	111	0	[85.71–85.71]	
Sadon_4_s	182	177	5	0	[97.25–97.25]		175	7	0	[96.15–96.15]	
Santiago_1_s	1359	1358	1	0	[99.93–99.93]	[99.83–99.86]	1356	3	0	[99.78–99.78]	[97.44–97.47]
Santiago_2_s	1164	1161	2	1	[99.74–99.83]		1135	28	1	[97.51–97.59]	
Santiago_3_s	777	777	0	0	[100–100]		735	42	0	[94.59–94.59]	
Santiago_4_s	182	180	2	0	[98.90–98.90]		167	15	0	[91.76–91.76]	
Warrilow_1_g	1359	1231	20	108	[90.58–98.40]	[91.13–98.63]	1226	24	109	[90.21–98.08]	[90.72–98.26]
Warrilow_2_g	1164	1059	13	92	[90.98–98.79]		1058	14	92	[90.89–98.69]	
Warrilow_3_g	777	716	11	50	[92.15–98.49]		712	14	51	[91.63–98.07]	
Warrilow_4_g	182	167	0	15	[91.76–100]		163	4	15	[89.56–97.60]	

Results were generated according to the workflow presented in Fig. 1. The first column lists the method name (see Table 1). The second column lists the number of analysed genomes per method. The next five columns list he number of genomes detected, the number of genomes not detected, the number of genomes where the outcome is unknown, the range between the more and the less conservative score for the in silico sensitivity per serotype per method, and the range between the more and the less conservative score for the in silico sensitivity averaged over the different serotypes per method (weighted for the different number of analysed genomes per serotype), when one mismatch was allowed at the 3′ end of primer-template pairs. The next five columns list the same information when no single mismatch was allowed at the 3′ end of primer-template pairs

For the in silico specificity, both intra- and interspecies specificity were evaluated. Intraspecies specificity was assessed by challenging all serotype-specific methods with all genomes belonging to the other serotypes (methods directed against the entire species cannot be evaluated as they are expected to pick up all serotypes). A more and a less conservative score were obtained for every serotype-specific RT-qPCR method by either excluding unknown cases, or including them as genomes not being detected (see Methods). Table 3 presents results when allowing for either one or no single mismatch in the last five bases of primer annealing sites at their 3′ end (all other thresholds were kept constant). When one mismatch in the last five bases of primer annealing sites at the 3′ end was allowed, five out of 11 methods obtained a perfect score of 100% for both the more and the less conservative score. This applies for the methods developed by Cecilia et al. [25], Kim et al. [33], Kong et al. [28], Laue et al. [34], and Santiago et al. [30]. The six remaining methods all also attain a score > 95% based on the less conservative score: Callahan et al. [31], Chien et al. [26], Ito et al. [27], Johnson et al. [7], Leparc-Goffart et al. [32], and Sadon et al. [29]. Notably, most wrong serotypes being detected appear to originate from RT-qPCR methods that target the third serotype, as found for the methods developed by Callahan et al. [31], Ito et al. [27], and Leparc-Goffart et al. [32]. When no single mismatch in the last five bases of primer annealing sites at the 3′ end was allowed, specificity for all methods for both the more and the less conservative score was > 99%. This indicates that most serotype-specific methods manage to discriminate with very high intraspecies specificity between the different serotypes, and that only few wrong serotypes are erroneously picked up. In particular, the third serotype might however suffer from false positives, warranting more scrutiny when developing methods that target this serotype. Interspecies specificity was obtained by challenging all RT-qPCR methods with whole genomes collected for West Nile virus from the NCBI Virus Variation Resource [12]. A more and a less conservative score were obtained for every RT-qPCR method by either excluding unknown cases, or including them as genomes not being detected (see Methods). Table 4 presents results when allowing for either one or no single mismatch in the last five bases of primer annealing sites at the 3′ end (all other thresholds were kept constant). All methods attain a perfect score of 100% for both the more and the less conservative score, both when allowing one or no single mismatch in the last five bases at the 3′ end, indicating extremely high interspecies specificity when using West Nile virus as an off-target species.

**Table 3:** Dengue virus RT-qPCR method performance in terms of intraspecies in silico specificity

Method name	# genomes analysed	One mismatch allowed at 3′ end of primer-template pairs					No mismatches allowed at 3′ end of primer-template pairs				
		# genomes detected	# genomes not detected	# genomes unknown	% in silico specificity	% average in silico specificity	# genomes detected	# genomes not detected	# genomes unknown	% in silico specificity	% average in silico specificity
Callahan_1_s	2123	0	2076	47	[100–100]	[95.43–95.48]	0	2076	47	[100–100]	[100–100]
Callahan_2_s	2318	0	2237	81	[100–100]		0	2237	81	[100–100]	
Callahan_3_s	2705	472	2233	0	[82.55–82.55]		0	2705	0	[100–100]	
Callahan_4_s	3300	0	3300	0	[100–100]		0	3300	0	[100–100]	
Cecilia_4_s	3300	0	1166	2134	[100–100]	[100–100]	0	1166	2134	[100–100]	[100–100]
Chien_1_s	2123	7	2116	0	[99.67–99.67]	[99.93–99.93]	0	2123	0	[100–100]	[100–100]
Chien_2_s	2318	0	2318	0	[100–100]		0	2318	0	[100–100]	
Chien_3_s	2705	0	2705	0	[100–100]		0	2705	0	[100–100]	
Chien_4_s	3300	0	3300	0	[100–100]		0	3300	0	[100–100]	
Ito_1_s	2123	0	1661	462	[100–100]	[99.12–99.46]	0	1661	462	[100–100]	[99.58–99.74]
Ito_2_s	2318	0	1037	1281	[100–100]		0	1037	1281	[100–100]	
Ito_3_s	2705	56	1822	827	[97.02–97.93]		27	1851	827	[98.56–99.00]	
Ito_4_s	3300	0	1779	1521	[100–100]		0	1779	1521	[100–100]	
Johnson_1_s	2123	1	2122	0	[99.95–99.95]	[99.99–99.99]	0	2123	0	[100–100]	[100–100]
Johnson_2_s	2318	0	2224	94	[100–100]		0	2224	94	[100–100]	
Johnson_3_s	2705	0	1138	1567	[100–100]		0	1138	1567	[100–100]	
Johnson_4_s	3300	0	2613	687	[100–100]		0	2613	687	[100–100]	
Kim_1_s	2123	0	1982	141	[100–100]	[100–100]	0	1982	141	[100–100]	[100–100]
Kim_2_s	2318	0	2312	6	[100–100]		0	2312	6	[100–100]	
Kim_3_s	2705	0	1488	1217	[100–100]		0	1488	1217	[100–100]	
Kim_4_s	3300	0	1060	2240	[100–100]		0	1060	2240	[100–100]	
Kong_1_s	2123	0	2123	0	[100–100]	[100–100]	0	2123	0	[100–100]	[100–100]
Kong_2_s	2318	0	2114	204	[100–100]		0	2114	204	[100–100]	
Kong_3_s	2705	0	2688	17	[100–100]		0	2688	17	[100–100]	
Kong_4_s	3300	0	3269	31	[100–100]		0	3269	31	[100–100]	
Laue_1_s	2123	0	1823	300	[100–100]	[100–100]	0	1823	300	[100–100]	[100–100]
Laue_2_s	2318	0	2316	2	[100–100]		0	2316	2	[100–100]	
Laue_3_s	2705	0	2695	10	[100–100]		0	2695	10	[100–100]	
Laue_4_s	3300	0	3176	124	[100–100]		0	3176	124	[100–100]	
Leparc_Goffart_1_s	2123	0	2114	9	[100–100]	[94.67–95.48]	0	2114	9	[100–100]	[100–100]
Leparc_Goffart_2_s	2318	0	742	1576	[100–100]		0	742	1576	[100–100]	
Leparc_Goffart_3_s	2705	472	2233	0	[82.55–82.55]		0	2705	0	[100–100]	
Leparc_Goffart_4_s	3300	0	3296	4	[100–100]		0	3296	4	[100–100]	
Sadon_1_s	2123	3	2119	1	[99.86–99.86]	[99.95–99.97]	3	2119	1	[99.86–99.86]	[99.95–99.97]
Sadon_2_s	2318	0	135	2183	[100–100]		0	135	2183	[100–100]	
Sadon_3_s	2705	0	146	2559	[100–100]		0	146	2559	[100–100]	
Sadon_4_s	3300	0	3300	0	[100–100]		0	3300	0	[100–100]	
Santiago_1_s	2123	0	2123	0	[100–100]	[100–100]	0	2123	0	[100–100]	[100–100]
Santiago_2_s	2318	0	2224	94	[100–100]		0	2224	94	[100–100]	
Santiago_3_s	2705	0	1138	1567	[100–100]		0	1138	1567	[100–100]	
Santiago_4_s	3300	0	2613	687	[100–100]		0	2613	687	[100–100]	

Results were generated according to the workflow presented in Fig. 1. The first column lists the method name (see Table 1). Only RT-qPCR methods for serotype-specific detection were evaluated. The second column lists the number of analysed genomes per method. The next five columns list the number of genomes detected, the number of genomes not detected, the number of genomes where the outcome is unknown, the range between the more and the less conservative score for the in silico specificity per serotype per method, and the range between the more and the less conservative score for the in silico specificity averaged over the different serotypes per method (weighted for the different number of analysed genomes per serotype), when one mismatch was allowed at the 3′ end of primer-template pairs. The next five columns list the same information when no single mismatch was allowed at the 3′ end of primer-template pairs

**Table 4:** Dengue virus RT-qPCR method performance in terms of interspecies in silico specificity

Method name	# genomes analysed	One mismatch allowed at 3′ end of primer-template pairs					No mismatches allowed at 3′ end of primer-template pairs				
		# genomes detected	# genomes not detected	# genomes unknown	% in silico specificity	% average in silico specificity	# genomes detected	# genomes not detected	# genomes unknown	% in silico specificity	% average in silico specificity
Callahan_1_s	927	0	861	66	[100–100]	[100–100]	0	861	66	[100–100]	[100–100]
Callahan_2_s	927	0	834	93	[100–100]		0	834	93	[100–100]	
Callahan_3_s	927	0	786	141	[100–100]		0	786	141	[100–100]	
Callahan_4_s	927	0	817	110	[100–100]		0	817	110	[100–100]	
Callahan_g	927	0	340	587	[100–100]	[100–100]	0	340	587	[100–100]	[100–100]
Cecilia_4_s	927	0	132	795	[100–100]	[100–100]	0	132	795	[100–100]	[100–100]
Chien_1_s	927	0	927	0	[100–100]	[100–100]	0	927	0	[100–100]	[100–100]
Chien_2_s	927	0	927	0	[100–100]		0	927	0	[100–100]	
Chien_3_s	927	0	927	0	[100–100]		0	927	0	[100–100]	
Chien_4_s	927	0	927	0	[100–100]		0	927	0	[100–100]	
Conceicao_g	927	0	3	924	[100–100]	[100–100]	0	3	924	[100–100]	[100–100]
Drosten_g	927	0	7	920	[100–100]	[100–100]	0	7	920	[100–100]	[100–100]
Gurukumar_g	927	0	67	860	[100–100]	[100–100]	0	67	860	[100–100]	[100–100]
Ito_1_s	927	0	0	927	[100–100]	[100–100]	0	0	927	[100–100]	[100–100]
Ito_2_s	927	0	10	917	[100–100]		0	10	917	[100–100]	
Ito_3_s	927	0	46	881	[100–100]		0	46	881	[100–100]	
Ito_4_s	927	0	10	917	[100–100]		0	10	917	[100–100]	
Johnson_1_s	927	0	113	814	[100–100]	[100–100]	0	113	814	[100–100]	[100–100]
Johnson_2_s	927	0	42	885	[100–100]		0	42	885	[100–100]	
Johnson_3_s	927	0	121	806	[100–100]		0	121	806	[100–100]	
Johnson_4_s	927	0	881	46	[100–100]		0	881	46	[100–100]	
Kim_1_s	927	0	52	875	[100–100]	[100–100]	0	52	875	[100–100]	[100–100]
Kim_2_s	927	0	788	139	[100–100]		0	788	139	[100–100]	
Kim_3_s	927	0	4	923	[100–100]		0	4	923	[100–100]	
Kim_4_s	927	0	871	56	[100–100]		0	871	56	[100–100]	
Kong_1_s	927	0	914	13	[100–100]	[100–100]	0	914	13	[100–100]	[100–100]
Kong_2_s	927	0	848	79	[100–100]		0	848	79	[100–100]	
Kong_3_s	927	0	913	14	[100–100]		0	913	14	[100–100]	
Kong_4_s	927	0	926	1	[100–100]		0	926	1	[100–100]	
Laue_1_s	927	0	927	0	[100–100]	[100–100]	0	927	0	[100–100]	[100–100]
Laue_2_s	927	0	927	0	[100–100]		0	927	0	[100–100]	
Laue_3_s	927	0	923	4	[100–100]		0	923	4	[100–100]	
Laue_4_s	927	0	822	105	[100–100]		0	822	105	[100–100]	
Leparc_Goffart_1_s	927	0	139	788	[100–100]	[100–100]	0	139	788	[100–100]	[100–100]
Leparc_Goffart_2_s	927	0	5	922	[100–100]		0	5	922	[100–100]	
Leparc_Goffart_3_s	927	0	848	79	[100–100]		0	848	79	[100–100]	
Leparc_Goffart_4_s	927	0	2	925	[100–100]		0	2	925	[100–100]	
Leparc_Goffart_g	927	0	359	568	[100–100]	[100–100]	0	359	568	[100–100]	[100–100]
Pongsiri_g	927	0	391	536	[100–100]	[100–100]	0	391	536	[100–100]	[100–100]
Sadon_1_s	927	0	3	924	[100–100]	[100–100]	0	3	924	[100–100]	[100–100]
Sadon_2_s	927	0	796	131	[100–100]		0	796	131	[100–100]	
Sadon_3_s	927	0	9	918	[100–100]		0	9	918	[100–100]	
Sadon_4_s	927	0	45	882	[100–100]		0	45	882	[100–100]	
Santiago_1_s	927	0	113	814	[100–100]	[100–100]	0	113	814	[100–100]	[100–100]
Santiago_2_s	927	0	42	885	[100–100]		0	42	885	[100–100]	
Santiago_3_s	927	0	121	806	[100–100]		0	121	806	[100–100]	
Santiago_4_s	927	0	881	46	[100–100]		0	881	46	[100–100]	
Warrilow_g	927	0	353	574	[100–100]	[100–100]	0	353	574	[100–100]	[100–100]

Results were generated according to the workflow presented in Fig. 1. The first column lists the method name (see Table 1). RT-qPCR methods for dengue virus detection (denoted by the extension ‘_g’) were evaluated by challenging them with 927 genomes of West Nile virus. RT-qPCR methods for dengue serotype-specific detection (denoted by the extension ‘_s’) were evaluated by challenging the primers and probe combination for every different serotype independently with 927 genomes of West Nile virus. The second column lists the number of analysed genomes per method. The next five columns list the number of genomes detected, the number of genomes not detected, the number of genomes where the outcome is unknown, the range between the more and the less conservative score for the in silico specificity per serotype per method, and the range between the more and the less conservative score for the in silico specificity averaged over the different serotypes per method (weighted for the different number of analysed genomes per serotype), when one mismatch was allowed at the 3′ end of primer-template pairs. The next five columns list the same information when no single mismatch was allowed at the 3′ end of primer-template pairs

## Discussion

We present, to the best of our knowledge, the first exhaustive comparison of routinely employed RT-qPCR methods for dengue virus detection, which will help to guide routine laboratories and policy makers towards selecting and implementing better suited methods and procedures. Our approach is novel because it provides an estimate for RT-qPCR method performance through an in silico evaluation of the appropriateness of primers and probes based on several thousands of dengue genomes, and was born from the need encountered by a routine enforcement laboratory to relatively quickly and easily screen large quantities of genome data in order to provide an estimate on the number of genomes in which the RT-qPCR method is expected to give a signal. This differs from currently existing tools for in silico primer design and evaluation that all have their own specific niches, but typically focus on the detailed investigation of distinct methods and do not allow the large-screen evaluation of different methods on several thousands of genomes. The trade-off to our approach is that it solely evaluates alignment statistics and therefore does not completely mimic the in vitro RT-qPCR reaction, which is influenced by a range of factors that are difficult or even impossible to account for in silico, such as running conditions (annealing time and temperature...), employed polymerase etc. [35]. This is why popular primer design and evaluation software packages will typically also take into consideration other factors such as melting temperatures, a balanced GC content, avoiding the formation of hair-pin structures as a consequence of self-complementarity etc. [36]. Our approach is hence specifically intended to screen primers and probe combinations on large quantities of genome data in order to evaluate their effectiveness for capturing their intended targeted genomic regions rather than creating and designing novel primers. We therefore envisage an approach where the aforementioned tools are employed to perform in-depth primer design and evaluation in the development process, combined with methods such as ours that offer the possibility to relatively quickly and easily screen large quantities of genome data in order to evaluate the feasibility of applying the RT-qPCR method on a larger set of samples than would be possible within the laboratory. For instance, the method developed by Chien et al. [26] was found to suffer from certain primer-template mismatches at the 3′ end, suggesting that introducing degeneracies at those specific locations could increase method performance (see Table 2, Additional file 1), which would be difficult to ascertain without using large amounts of genome data. Additionally, we found that the methods developed by Callahan et al. [31], Ito et al. [27], and Leparc-Goffart et al. [32], may suffer from a reduced intraspecies specificity for the third serotype (see Table 3). Although both these observations need to be experimentally validated and no in silico method will ever manage to replace the important process of experimental validation, results presented in Tables 2, 3 and 4 are based on the screening of more than 3000 dengue virus and almost 1000 West Nile virus genomes, in contrast to the limited set of samples traditionally employed for experimental validation [9]. This illustrates how our in silico large-scale screening can be used to complement the traditional RT-qPCR method development process.

Employing suitable threshold values for the different in silico selection criteria is important to ensure proper scoring of method performance. Values for the first (i.e. maximum mismatch percentage over the entire annealing site of the primer/probe) and second (i.e. minimum length of annealing site relative to total primer/probe length) selection criteria were put at 20% and 80%, respectively, in order to comply with previous observations from the literature. Christopherson et al. [17] found that for a viral case study (HIV), six mismatches over a primer length of 30 residues (20%) drastically reduced PCR yield. Lefever et al. [18] observed that, by means of creating synthetic templates and primers to assess the effect of mismatches in primer annealing sites on qPCR assay performance, a number of four mismatches over a primer length of 20 residues (20%) completely blocked the reaction. The threshold value for the first criterion simulates this effect for primers and probes of variable lengths by not allowing more than 20% of mismatches, while the threshold value for the second criterion enforces that the first criterion is evaluated over a region long enough of at least 80% for valid interpretation. Threshold values for the third selection criterion (i.e. the number of allowed mismatched bases for primer-template pairs at the five last bases at their 3′ end) were similarly chosen based on previous research that indicated that even a small number of mismatches in this region can strongly influence the reaction [10]. Kwok et al. [19] for instance observed that for a viral case study (HIV), a single mismatch at the 3′ end of the primer-template pair negatively affected PCR yield with variable degrees dependent upon the specific substitution, whereas two or more mismatches drastically reduced PCR yield, which is why results in Tables 2, 3 and 4 are always presented allowing both for either one or no single mismatch in this region.

RT-qPCR method performance is typically expressed in terms of its sensitivity (i.e. the ability of the method to detect a wide range of targets by a defined relatedness percentage) and its specificity (i.e. the ability of the method to distinguish the target from similar but genetically distinct non-targets), which are also referred to in this context as inclusivity and exclusivity ([37, 38], see also Methods), and are obtained by challenging the method with a set of a priori known target and off-target samples. Obtaining high method sensitivity is imperative to ensure dengue virus infections will be correctly picked up, but adequate method specificity is also important as this ensures off-target organisms will not falsely be identified as dengue virus. Table 2 describes performance in terms of in silico sensitivity, whereas Tables 3 and 4 describe performance in terms of intra- and interspecies in silico specificity, respectively. Both intra- and interspecies specificity were generally found to be very high for all RT-qPCR methods, whereas sensitivity displayed more variation between the different methods. When not allowing a single mismatch in the last five bases at the 3′ end of primer-template pairs, only three out of eighteen methods obtained an in silico sensitivity > 95% based on the conservative score. Even when allowing for one such mismatch, only nine out of eighteen methods obtained an in silico sensitivity > 95 based on the conservative score. The difference in performance when allowing one or no single mismatch in the last five bases at the 3′ end of primer-template pairs, indicates that this could represent a widespread issue for dengue virus RT-qPCR detection methods that merits more attention in their development process, especially in light of the many studies that have highlighted the detrimental effect thereof [15, 16, 18, 19, 39]. This could suggest that for dengue RT-qPCR methods, some specificity could potentially be sacrificed in order to obtain higher sensitivity, for instance by introducing degeneracies at problematic primer positions to alleviate this effect, although these suggestions can only be validated through experiments in the laboratory.

Our approach has been made available as a public tool to enable evaluating RT-qPCR method performance for other viral species to be used by laboratories that do not have access to the required bioinformatics expertise to perform such analyses (see Methods). In particular, the thresholds values for the selection criteria employed in this study (see Fig. 1) can be modified by the user to be more or less strict dependent upon the desired application. Our approach can therefore easily be extrapolated to other important (re-)emerging viral pathogens that pose a public health threat and for which whole genome data is available. As more genomic data will become available in the future, the availability and development of such novel methods that can incorporate these data for large-scale screening will aid to keep evaluating and improving RT-qPCR method performance.

## Conclusions

The detection of viral infection is an important public health topic, since it allows providing appropriate disease treatment for infected individuals, but also taking appropriate measures aiming to contain and prevent viral spread in afflicted regions. Diagnosis is often performed through RT-qPCR methods that imply an accurate design of both primers and probe to ensure adequate performance, whereas routinely employed RT-qPCR methods were traditionally constructed based on a limited set of reference samples that may not be representative for the entire population. We presented a proof-of-concept approach that allows to incorporate screening of large-scale genomic information into the evaluation of RT-qPCR method performance, by recovering the targeted genomic regions and evaluating whether annealing sites are adequately conserved to result in a signal. Though based completely on an in silico workflow, this provides a proxy for RT-qPCR method effectiveness that can be used in the development and evaluation process of RT-qPCR methods in combination with the traditional laboratory validation on reference samples.

## Methods

### Collection of whole genome data

The NCBI Dengue Virus Variation Resource available at http://www.ncbi.nlm.nih.gov/genome/viruses/variation/dengue [12] was mined for unique full-length (including the 5’UTR and 3’ UTR regions) nucleotide sequences for all serotypes (allowing for any disease, host, region/country, and isolation source) on the 18th of August 2016. In total, 1359, 1164, 777, and 182 genomes were collected for serotypes 1, 2, 3 and 4, respectively (no genomes were available for the fifth serotype). Similarly, the NCBI West Nile Virus Variation Resource available at https://www.ncbi.nlm.nih.gov/genomes/VirusVariation/Database/nph-select.cgi?taxid=11082 [12] was mined for unique full-length (including the 5’UTR and 3’ UTR regions) nucleotide sequences (allowing for any host, region/country, and isolation source) on the 11th of July 2018. In total, 927 genomes were collected. All genome sequences for these species used in this study, are available at the following location: https://github.com/BioinformaticsPlatformWIV-ISP/SCREENED/blob/master/inputSCREENED.zip (see also Additional file 1).

### Collection of RT-qPCR methods

Eighteen RT-qPCR dengue virus detection methods were collected from the literature (see Table 1). The following nomenclature was adapted: surname of the first author of the publication, an underscore followed by the serotype under evaluation, followed by ‘s’ or ‘g’ denoting whether the method was developed originally for serotyping (i.e. detecting only one specific serotype) or dengue virus detection (i.e. detecting the species, including all four serotypes), respectively. Note that the method developed by Cecilia et al. [25] was specifically developed only for the fourth serotype. Additional file 1: Table S1 lists detailed sequence information extracted from each corresponding publication for the forward and reverse primers, and probe. A template reference sequence for the targeted genomic region was obtained manually for every RT-qPCR method through aligning the primers and probe sequences to selected dengue virus reference genomes using BLAST. Both the accession numbers of these reference genomes, and the extracted template reference sequences, are available in Additional file 1: Table S1. All sequence information for the evaluated methods employed in this study, is also available at the following location: https://github.com/BioinformaticsPlatformWIV-ISP/SCREENED/blob/master/inputSCREENED.zip (see also Additional file 1).

### Workflow for in silico method evaluation

#### Recovery of targeted genomic regions through a two-step BLAST approach

Figure 1 provides an overview of the workflow employed for evaluating RT-qPCR methods. A two-step BLAST approach was used by first extracting the targeted genomic regions from the genomes, and afterwards investigating the hybridisation properties of the recovered regions. This two-step approach was motivated by the observation that directly aligning short oligonucleotides (i.e. primers and probes) against whole genomes typically results in a long list of hits with varying degrees of sequence similarity. However, primers and probes do not simply need to anneal to the genome, they also need to have a specific orientation in respect to each other to result in a signal: the forward and reverse primers need to be within the vicinity of each other, they need to be directed towards each other, and the probe needs to be situated between both primers. For instance, even if the forward and reverse primers anneal to the genome within a distance close enough and with an orientation directed towards each other, a signal will not be generated if the probe does not anneal to the resulting PCR product. Tools intended for in-depth evaluation and construction of novel primer combinations will also incorporate additional information on, amongst others, melting temperatures, GC content, and avoiding self-complementarity and hairpin structures, in order to narrow down the list of potential targets. Such analyses are however complex and computationally intensive and therefore not suited for screening thousands of genomes. By utilizing a template reference for the targeted genomic region and first extracting it in the genome under investigation, the requirement for a proper orientation of primers and probes is respected while the computational burden and complexity of a more extended analysis is efficiently mitigated. The second BLAST step nevertheless ensures a thorough evaluation by ensuring that a minimal set of hybridization criteria is respected. The BLAST algorithm was used for both steps because it has been previously shown to be extremely sensitive, but does suffer from the possibility that an incomplete alignment is returned because BLAST is based on a local alignment strategy that can have trouble with recovering the ends of aligned regions through mismatches [15]. An extension of the local alignment was therefore always applied to correct for this (see Additional file 1).

In the first step, the BLASTN program [40] from the BLAST suite (v2.2.30) was used to detect the specific sequence of the targeted region in each analysed genome; by employing the template reference sequence (see above) as query, and the entire genomic sequence as subject. The following BLASTN settings were used (all other options were left at their default values): ‘-max_target_seqs 1’, ‘-strand plus’, ‘-reward 1’, and ‘-penalty − 1’. Reward and penalty scores for nucleotide matches and mismatches, respectively, were deliberately not put too stringent to account for the strong natural variation in viral populations. Recovered hits were sorted based on their bit score, and the best scoring hit was taken as the recovered targeted genomic region. Although this logic may be violated through the recovery of wrong or shorter sequences in the investigated genome, imposed selection criteria (see below) will ensure that such cases are not falsely propagated. In case no such region could be extracted, the genome was classified as unknown because this could either be due to the genome not containing the targeted region, or alternatively the genomic sequence being incomplete.

In the second step, the BLASTN-SHORT program from the BLAST suite was used to detect the annealing sites targeted by the primers and probe in the recovered region of the genome under investigation; by employing the primer/probe sequence as query, and the recovered targeted genomic region as subject. The following BLASTN-SHORT settings were set (all other options were left at their default values): ‘-max_target_seqs 1’, ‘-strand plus’, ‘-reward 1’, ‘-penalty − 1’, and ‘-word_size 4’. The sequence of the reverse primer was always reverse complemented to ensure both sequences have the same orientation. Reward and penalty scores for nucleotide matches and mismatches, respectively, and word size, were deliberately not set too stringent to account for the strong natural variation in viral populations. Recovered hits were sorted based on their bit score, and the best scoring hit was considered to represent the annealing site. Although this logic may be violated through the recovery of wrong or shorter sequences, imposed selection criteria (see below) will prevent these hits from being falsely propagated. Additionally, the search space for primers and probes is limited to only the recovered targeted genomic sequence, guarding against an overflow of hits throughout the remainder of the genome. For methods with degenerate nucleotide characters within their primer(s) and/or probe sequence(s), all possible sequence variants were evaluated using the approach above, and then the best scoring variant was selected.

#### Criteria to test whether a (theoretical) signal is generated

Three logical checks assessed whether primers and probe combinations for every method were similar enough to their corresponding target region in the analysed genome to allow annealing and hence detection. First, the mismatch score between all primers and probes and their recovered target sites (based on the total alignment length, but accounting for nucleotide degeneracies), should be lower than a predefined cut-off that was set at 20% for all analyses. Second, the total alignment length of all primers and probes relative to their total length, should be higher than a predefined cut-off, which was set at 80% for all analyses. Third, for the last five bases at the 3′ end of the forward and reverse primers, there should be no more mismatches than a predefined cut-off, which was set either at one or zero bases. Threshold values for these criteria were selected based on observations from the literature (see Discussion). Passing all three criteria was required in order for the analysed genome to be considered as detected by the RT-qPCR method. Genomes not passing these criteria were subdivided into two classes dependent upon the position of the targeted genomic region. Genomes where this region was located at either the end or beginning of the genomic sequence were considered as unknown cases because this could indicate either that the genomic sequence was incomplete at its boundaries, or alternatively that the genome does not contain the full targeted genomic region. Otherwise, the genome was considered as not detected by the RT-qPCR method.

#### Scoring method performance by means of in silico sensitivity

Sensitivity is defined as the ability of a method to detect a wide range of targets by a defined relatedness, also referred to in this context as inclusivity [38], and is widely used to evaluate assay performance (e.g. [37]). For in silico assays, sensitivity has also been defined as the likelihood that an assay will detect a sequence variation when present within the analysed genome [41], which was extended to our work as the likelihood that a RT-qPCR method under investigation will properly detect the correct serotype and/or species. Since this evaluation is qualitative (i.e. the genome is detected by the method or not) rather than quantitative at a certain limit of detection such as is typically taken into consideration for most laboratory validations [42], this performance characteristic can be considered to correspond with the diagnostic sensitivity of the assay [43]. Since the assay is completely based on an in silico approach, we therefore denoted this as the ‘% in silico sensitivity’. This metric was obtained by challenging all 18 RT-qPCR methods (see Table 1) with all genomes of the corresponding serotype. Methods developed for dengue virus detection without serotype discrimination (denoted by ‘_g’, see above), were still analysed for all four serotypes separately to facilitate recognition of serotypes that might exhibit deviant behaviour. The metric was then calculated by taking the ratio of the total number of genomes that led in silico to detection divided by the total number of analysed genomes. A more and a less conservative score were always calculated by either including unknown cases (see Fig. 1) as genomes not being detected, or excluding them as genomes where the genomic sequence is incomplete so that they were considered as missing data (see also Additional file 1). This resulted in a range for the ‘% in silico sensitivity’ for every method:

$$ \%\mathrm{in}\ \mathrm{silico}\ \mathrm{sensitivity}=\left[\ \frac{\#\mathrm{genomes}\ \mathrm{detected}}{\#\mathrm{genomes}\ \mathrm{analyzed}} - \frac{\#\mathrm{genomes}\ \mathrm{detected}}{\#\mathrm{genomes}\ \mathrm{analyzed}-\#\mathrm{genomes}\ \mathrm{unknown}}\right] $$

A weighted average for the ‘% in silico sensitivity’ was then calculated for every method by taking the average of its four analysed serotypes, weighted for the total number of analysed genomes per serotype. Results thereof are presented in Table 2.

#### Scoring method performance by means of in silico intra- and interspecies specificity

Specificity is defined as the ability of a method to distinguish the target from similar but genetically distinct non-targets [38], and is also widely used to evaluate assay performance (e.g. [37]). For in silico assays, specificity has also been defined as the likelihood that an assay will not detect a sequence variation when not present within the analysed genome [41], which was extended to our work as the likelihood that a RT-qPCR method under investigation will not incorrectly detect a dengue species and/or serotype when challenged with non-target genomes. As for sensitivity, this evaluation is qualitative rather than quantitative, and therefore corresponds with the diagnostic specificity of the assay [43]. Since the assay is completely based on an in silico approach, we therefore denoted this as the ‘% in silico specificity’. This metric was obtained at the intraspecies level by challenging all serotype-specific RT-qPCR methods (denoted by ‘_s’, see above) with all genomes belonging to the three other serotypes. RT-qPCR methods designed for dengue virus detection (denoted by ‘_g’) cannot be considered as they are expected to pick up all serotypes. Similarly, this metric was obtained at the interspecies level by challenging all RT-qPCR methods with genomes belonging to West Nile virus, which is also a member of the *Flavivirus* genus but a different species [44] and therefore ideally suited as a genetically similar but distinct non-target. The metric was then calculated by taking the ratio of the total number of genomes that led in silico not to detection divided by the total number of analysed genomes. Although it is to be expected that very specific methods will result in many unknown cases through the targeted genomic region not being present in the genome, a more and a less conservative score were nevertheless always calculated by either excluding unknown cases as missing data (see also Additional file 1), or including them as genomes not being detected. This results in a range for the ‘% in silico specificity’ for every method:

$$ \%\mathrm{in}\ \mathrm{silico}\ \mathrm{specificity}=\left[\ \frac{\#\mathrm{genomes}\ \mathrm{not}\ \mathrm{detected}}{\#\mathrm{genomes}\ \mathrm{not}\ \mathrm{detected}+\#\mathrm{genomes}\ \mathrm{detected}} - \frac{\#\mathrm{genomes}\ \mathrm{not}\ \mathrm{detected}+\#\mathrm{genomes}\ \mathrm{unknown}}{\#\mathrm{genomes}\ \mathrm{analyzed}}\right] $$

A weighted average for the ‘% in silico specificity’ was then calculated for every method by taking the average of its four analysed serotypes, weighted for the total number of analysed genomes per serotype. Results thereof are presented in Tables 3 and 4 for intra- and interspecies specificity, respectively.

### Availability and requirements

Our approach has been made available as a public web tool named ‘polymeraSe Chain Reaction Evaluation through largE-scale miNing of gEnomic Data’ or simply SCREENED, using the Galaxy Workflow Management system [45], and can be accessed at https://galaxy.sciensano.be. The tool requires the user to specify an input file containing all genomes to be analysed (in FASTA format), an input file containing all the sequence information for the primers and probe(s), and a template reference for the targeted genomic region, for every method under evaluation (in tab-delimited format similar to Additional file 1: Table S1). Output consists of a detailed output file containing the sequences of recovered targeted genomic regions and their primer and probe annealing sites, and results of selection criteria, for all genomes; and a summary output file containing all genomes that are detected. More advanced options, such as specific threshold values for the selection criteria to be used to investigate their effect on the output, can also be set. A full tutorial that takes the user step-by-step through the tool is also available (see Additional file 1). Our approach can also be run directly on the command line for more expert users by means of the source code (see ‘Availability of data and materials’).

## Additional file

Additional file 1: Supplementary table, supporting data, and supporting information. (DOCX 1229 kb)

## Abbreviations

BLAST: Basic Local Alignment Search Tool
HIV: Human Immunodeficiency Virus
HTS: High-Throughput Sequencing
NGS: Next-Generation Sequencing
RNA: RiboNucleic Acid
RT-qPCR: quantitative reverse transcriptase real-time polymerase chain reaction
SCREENED: polymeraSe Chain Reaction Evaluation through largE-scale miNing of gEnomic Data
UTR: UnTranslated Region
WGS: Whole Genome Sequencing
