# Supplementary material for: Application of whole genome data for in silico evaluation of primers and probes routinely employed for the detection of viral species by RT-qPCR using dengue virus as a case study
Source: BMC Bioinformatics. 2018 Sep 4;19:312. doi: 10.1186/s12859-018-2313-0 (PMC6123964; doi:10.1186/s12859-018-2313-0)
Supplement: Supplementary file 1 — Supplementary table, supporting data, and supporting information. (DOCX 1229 kb) [file 12859_2018_2313_MOESM1_ESM.docx]

Additional file 1

**Application of whole genome data for *in silico* evaluation of primers and probes routinely employed for the detection of viral species by RT-qPCR using dengue virus as a case study**

Kevin Vanneste^1,*,†^, Linda Garlant^1,*^, Sylvia Broeders^1,#^, Steven Van Gucht^2^, Nancy H. Roosens^1, †^

^1^ Transversal & Applied Genomics, Sciensano, Brussels (1050), Belgium

^2^ Viral Diseases, Sciensano, Brussels (1050), Belgium

* Contributed equally to this study

^#^ Current address: Quality of Laboratories, Sciensano, Brussels (1050), Belgium

^†^ Corresponding authors

E-mail addresses of authors: kevin.vanneste@sciensano.be, linda.garlant@gmail.com, sylvia.broeders@sciensano.be, steven.vangucht@sciensano.be, nancy.roosens@sciensano.be

## Table of contents

[Table of contents 2](#_Toc520970117)

[Supplementary Table S1: Detailed sequence information for the 18 RT-qPCR methods evaluated in this study for dengue virus detection 3](#_Toc520970118)

[Supporting data: Input and output data employed and generated in this study 10](#_Toc520970119)

[Input data 10](#_Toc520970120)

[Output data 10](#_Toc520970121)

[Supporting information: Extension of local alignments retrieved by BLAST 12](#_Toc520970122)

[Supporting information: Unknown cases most likely represent incomplete genomic sequences 16](#_Toc520970123)

[Supporting information: The low *in silico* performance of the method developed by Chien et al. can be explained by primer mismatches in the last five bases at their 3’ end 20](#_Toc520970124)

[Supporting information: Tutorial for using SCREENED 22](#_Toc520970125)

[Objective and purpose 22](#_Toc520970126)

[Accessing Galaxy 22](#_Toc520970127)

[Uploading data 23](#_Toc520970128)

[Running SCREENED 25](#_Toc520970129)

[Viewing and downloading output 26](#_Toc520970130)

[Analysing output 27](#_Toc520970131)

[Additional options and analyses 28](#_Toc520970132)

[References 31](#_Toc520970133)

## Table S1: Detailed sequence information for the 18 RT-qPCR methods evaluated in this study for dengue virus detection

| **Method name** | **Reference genome NCBI accession number** | **Forward primer** | **Reverse primer** | **Probe** | **Template reference for the targeted genomic region** |
| --- | --- | --- | --- | --- | --- |
| Callahan_1_g | NC_001477.1 | AAGGACTAGAGGTTAKAGGAGACCC | GGCGYTCTGTGCCTGGAWTGATG | AACAGCATATTGACGCTGGGAGAGACC | AAGGACTAGAGGTTAGAGGAGACCCCCCGCACAACAACAAACAGCATATTGACGCTGGGAGAGACCAGAGATCCTGCTGTCTCTACAGCATCATTCCAGGCACAGAACGCC |
| Callahan_2_g | NC_001474.2 | AAGGACTAGAGGTTAKAGGAGACCC | GGCGYTCTGTGCCTGGAWTGATG | AACAGCATATTGACGCTGGGAAAGACC | AAGGACTAGAGGTTAGAGGAGACCCCCCCGAAACAAAAAACAGCATATTGACGCTGGGAAAGACCAGAGATCCTGCTGTCTCCTCAGCATCATTCCAGGCACAGAACGCC |
| Callahan_3_g | NC_001475.2 | AAGGACTAGAGGTTAKAGGAGACCC | GGCGYTCTGTGCCTGGAWTGATG | AACAGCATATTGACGCTGGGAGAGACC | AAGGACTAGAGGTTAGAGGAGACCCCCCGCAAATAAAAACAGCATATTGACGCTGGGAGAGACCAGAGATCCTGCTGTCTCCTCAGCATCATTCCAGGCACAGAACGCC |
| Callahan_4_g | NC_002640.1 | AAGGACTAGAGGTTAKAGGAGACCC | GGCGYTCTGTGCCTGGAWTGATG | AACAGCATATTGACGCTGGGAAAGACC | AGGACTAGAGGTTAGAGGAGACCCCCCCAACACAAAAACAGCATATTGACGCTGGGAAAGACCAGAGATCCTGCTGTCTCTGCAACATCAATCCAGGCACAGAGCGCC |
| Callahan_1_s | NC_001477.1 | GACACCACACCCTTTGGACAA | CACCTGGCTGTCACCTCCAT | AGAGGGTGTTTAAAGAGAAAGTTGACACGCG | GACACCACACCCTTTGGACAACAGAGGGTGTTTAAAGAGAAAGTTGACACGCGTACACCAAAAGCGAAACGAGGCACAGCACAAATTATGGAGGTGACAGCCAGGTG |
| Callahan_2_s | NC_001474.2 | CATGGCCCTKGTGGCG | CCCCATCTYTTCAGTATCCCTG | TCCTTCGTTTCCTAACAATCC | CATGGCCCTGGTGGCGTTCCTTCGTTTCCTAACAATCCCACCAACAGCAGGGATATTGAAGAGATGGGG |
| Callahan_3_s | NC_001475.2 | GGGAAAACCGTCTATCAATA | CGCCATAACCAATTTCATTGG | CACAGTTGGCGAAGAGATTCTCAAGAGGA | GGGAAAACCGTCTATCAATATGCTGAAACGCGTGAGAAACCGTGTGTCAACTGGATCACAGTTGGCGAAGAGATTCTCAAAAGGACTGCTGAACGGCCAGGGACCAATGAAATTGGTTATGGCG |
| Callahan_4_s | NC_002640.1 | TGAAGAGATTCTCAACCGGAC | AATCCCTGCTGTTGGTGGG | TCATCACGTTTTTGCGAGTCCTTTCCA | TGAAGAGATTCTCAACCGGACTTTTTTCTGGGAAAGGACCCTTACGGATGGTGCTAGCATTCATCACGTTTTTGCGAGTCCTTTCCATCCCACCAACAGCAGGGATT |
| Chien_1_s | NC_001477.1 | TACAACATGATGGGAAAGCGAGAGAAAAA | GTGTCCCAGCCGGCGGTGTCATCAGC | TCAGAGACATATCAAAGATTCCAGGGGG | TACAACATGATGGGAAAGAGAGAGAAAAAATTAGGAGAGTTCGGAAAGGCAAAAGGAAGTCGCGCAATATGGTACATGTGGTTGGGAGCGCGCTTTTTAGAGTTTGAAGCCCTTGGTTTCATGAATGAAGATCACTGGTTCAGCAGAGAGAATTCACTCAGTGGAGTGGAAGGAGAAGGACTCCACAAACTTGGATACATACTCAGAGACATATCAAAGATTCCAGGGGGAAATATGTATGCAGATGACACAGCCGGATGGGACAC |
| Chien_2_s | NC_001474.2 | TACAACATGATGGGAAAGCGAGAGAAAAA | GTGTCCCAGCCGGCGGTGTCATCAGC | AAGAGACGTGAGCAGGAAGGAAGGGGGAGC | TACAACATGATGGGAAAAAGAGAGAAGAAGCTAGGGGAATTCGGCAAGGCAAAAGGCAGCAGAGCCATATGGTACATGTGGCTTGGAGCACGCTTCTTAGAGTTTGAAGCCCTAGGATTCTTAAATGAAGATCACTGGTTCTCCAGAGAGAACTCCCTGAGTGGAGTGGAAGGAGAAGGGCTGCACAAGCTAGGTTACATTCTAAGAGACGTGAGCAAGAAAGAGGGAGGAGCAATGTATGCCGATGACACCGCAGGATGGGATAC |
| Chien_3_s | NC_001475.2 | TACAACATGATGGGAAAGCGAGAGAAAAA | GTGTCCCAGCCGGCGGTGTCATCAGC | TGAGAGATATTTCCAAGATACCCGGAGGAG | TATAACATGATGGGCAAGAGAGAGAAAAAACTTGGAGAGTTTGGCAAAGCAAAAGGCAGTAGAGCTATATGGTACATGTGGTTGGGAGCCAGGTACCTTGAGTTCGAAGCCCTTGGATTCTTAAATGAAGACCACTGGTTCTCGCGTGAAAACTCTTACAGTGGAGTAGAAGGAGAAGGACTGCACAAGCTAGGCTACATATTAAGGGACATTTCCAAGATACCCGGAGGAGCCATGTATGCTGATGACACAGCTGGTTGGGACAC |
| Chien_4_s | NC_002640.1 | TACAACATGATGGGAAAGCGAGAGAAAAA | GTGTCCCAGCCGGCGGTGTCATCAGC | TGGAGGAGATAGACAAGAAGGATGGAGACC | TATAACATGATGGGAAAACGTGAGAAAAAGTTAGGAGAGTTTGGCAGAGCCAAGGGAAGCCGAGCAATCTGGTACATGTGGCTGGGAGCGCGGTTTCTGGAATTTGAAGCCCTGGGTTTTTTGAATGAAGATCACTGGTTTGGCAGAGAAAATTCATGGAGTGGAGTGGAAGGGGAAGGTCTGCACAGATTGGGATATATCCTGGAGGAGATAGACAAGAAGGATGGAGACCTAATGTATGCTGATGACACAGCAGGCTGGGACAC |
| Conceicao_1_g | NC_001477.1 | ATTAGAGAGCAGATCTCTG | TGACACGCGGTTTC | TCAATATGCTGAAACGCG | ATTAGAGAGCAGATCTCTGATGAACAACCAACGGAAAAAGACGGGTCGACCGTCTTTCAATATGCTGAAACGCGCGAGAAACCGCGTGTCA |
| Conceicao_2_g | NC_001474.2 | ATTAGAGAGCAGATCTCTG | TGACACGCGGTTTC | TCAATATGCTGAAACGCG | ATTAGAGAGCAGATCTCTGATGAATAACCAACGGAAAAAGGCGAAAAACACGCCTTTCAATATGCTGAAACGCGAGAGAAACCGCGTGTCG |
| Conceicao_3_g | NC_001475.2 | ATTAGAGAGCAGATCTCTG | TGACACGCGGTTTC | TCAATATGCTGAAACGCG | ATTAGAGAGCAGATCTCTGATGAACAACCAACGGAAGAAGACGGGAAAACCGTCTATCAATATGCTGAAACGCGTGAGAAACCGTGTGTCA |
| Conceicao_4_g | NC_002640.1 | ATTAGAGAGCAGATCTCTG | TGACACGCGGTTTC | TCAATATGCTGAAACGCG | AATAGAGAGCAGATCTCTGGAAAAATGAACCAACGAAAAAAGGTGGTTAGACCACCTTTCAATATGCTGAAACGCGAGAGAAACCGCGTATCA |
| Drosten_1_g | NC_001477.3 | GGATAGACCAGAGATCCTGCTGT | CAWTCCATYTTSYGGCGYTC | CAGCATCATTCCAGGCACAG | GGAGAGACCAGAGATCCTGCTGTCTCTACAGCATCATTCCAGGCACAGAACGCCAAAAAATGGAATG |
| Drosten_2_g | NC_001474.4 | GGATAGACCAGAGATCCTGCTGT | CAWTCCATYTTSYGGCGYTC | CAGCATCATTCCAGGCACAG | GGAAAGACCAGAGATCCTGCTGTCTCCTCAGCATCATTCCAGGCACAGAACGCCAGAAAATGGAATG |
| Drosten_3_g | NC_001475.4 | GGATAGACCAGAGATCCTGCTGT | CAWTCCATYTTSYGGCGYTC | CAGCATCATTCCAGGCACAG | GGAGAGACCAGAGATCCTGCTGTCTCCTCAGCATCATTCCAGGCACAGAACGCCAGAAAATGGAATG |
| Drosten_4_g | NC_002640.3 | GGATAGACCAGAGATCCTGCTGT | CAWTCCATYTTSYGGCGYTC | CAGCATCATTCCAGGCACAG | GGAAAGACCAGAGATCCTGCTGTCTCTGCAACATCAATCCAGGCACAGAGCGCCGCAAGATGGATTG |
| Gurukumar_1_g | NC_001477.1 | GARAGACCAGAGATCCTGCTGTCT | ACCATTCCATTTTCTGGCGTT | AGCATCATTCCAGGCAC | AGACCAGAGATCCTGCTGTCTCTACAGCATCATTCCAGGCACAGAACGCCAAAAAATGGAATGGT |
| Gurukumar_2_g | NC_001474.2 | GARAGACCAGAGATCCTGCTGTCT | ACCATTCCATTTTCTGGCGTT | AGCATCATTCCAGGCAC | GAAAGACCAGAGATCCTGCTGTCTCCTCAGCATCATTCCAGGCACAGAACGCCAGAAAATGGAATGGT |
| Gurukumar_3_g | NC_001475.2 | GARAGACCAGAGATCCTGCTGTCT | ACCATTCCATTTTCTGGCGTT | AGCATCATTCCAGGCAC | GAGAGACCAGAGATCCTGCTGTCTCCTCAGCATCATTCCAGGCACAGAACGCCAGAAAATGGAATGGT |
| Gurukumar_4_g | NC_002640.1 | GARAGACCAGAGATCCTGCTGTCT | ACCATTCCATTTTCTGGCGTT | AGCATCATTCCAGGCAC | GAAAGACCAGAGATCCTGCTGTCTCTGCAACATCAATCCAGGCACAGAGCGCCGCAAGATGGATTGGT |
| Cecilia_4_s | NC_002640.1 | AAGCCAGGAGGAAGCTGTACTCCT | CAATCCATCTTGCGGCGCTCT | CTGTCTCTGCAACATCAATCCAGGCA | AAGCCAGGAGGAAGCTGTACTCCTGGTGGAAGGACTAGAGGTTAGAGGAGACCCCCCCAACACAAAAACAGCATATTGACGCTGGGAAAGACCAGAGATCCTGCTGTCTCTGCAACATCAATCCAGGCACAGAGCGCCGCAAGATGGATTG |
| Ito_1_s | NC_001477.1 | GAACATGGRACAAYTGCAACYAT | CCGTAGTCDGTCAGCTGTATTTCA | ACACCTCAAGCTCC | GAACATGGAACAACTGCAACCATAACACCTCAAGCTCCCACGTCGGAAATACAGCTGACAGACTACGG |
| Ito_2_s | NC_001474.2 | ACACCACAGAGTTCCATCACAGA | CATCTCATTGAAGTCNAGGCC | CGATGGARTGCTCTC | ACACCACAGAGTTCCATCACAGAAGCAGAATTGACAGGTTATGGCACTGTCACAATGGAGTGCTCTCCAAGAACGGGCCTCGACTTCAATGAGATG |
| Ito_3_s | NC_001475.2 | ATGAGATGYGTGGGAGTRGGAAAC | CACCACDTCAACCCACGTAGCT | AGATTTTGTGGAAGGYCT | ATGAGATGTGTGGGAGTAGGAAACAGAGATTTTGTGGAAGGGCTATCAGGAGCTACGTGGGTTGACGTGGTG |
| Ito_4_s | NC_002640.1 | GGTGACRTTYAARGTHCCTCAT | WGARTGCATRGCTCCYTCCTG | CCAAGAGACAGGATGTGACAGTGCTRGGATC | GGTGACATTTAAGGTTCCTCATGCCAAGAGACAGGATGTGACAGTGCTGGGATCTCAGGAAGGAGCCATGCATTCT |
| Johnson_1_s | NC_001477.1 | CAAAAGGAAGTCGTGCAATA | CTGAGTGAATTCTCTCTACTGAACC | CATGTGGTTGGGAGCACGC | CAAAAGGAAGTCGCGCAATATGGTACATGTGGTTGGGAGCGCGCTTTTTAGAGTTTGAAGCCCTTGGTTTCATGAATGAAGATCACTGGTTCAGCAGAGAGAATTCACTCAG |
| Johnson_2_s | NC_001474.2 | CAGGTTATGGCACTGTCACGAT | CCATCTGCAGCAACACCATCTC | CTCTCCGAGAACAGGCCTCGACTTCAA | CAGGTTATGGCACTGTCACAATGGAGTGCTCTCCAAGAACGGGCCTCGACTTCAATGAGATGGTGTTGCTGCAGATGG |
| Johnson_3_s | NC_001475.2 | GGACTGGACACACGCACTCA | CATGTCTCTACCTTCTCGACTTGTCT | ACCTGGATGTCGGCTGAAGGAGCTTG | GGACTGGACACACGCACCCAAACCTGGATGTCGGCTGAAGGAGCTTGGAGACAAGTCGAGAAGGTAGAGACATG |
| Johnson_4_s | NC_002640.1 | TTGTCCTAATGATGCTGGTCG | TCCACCTGAGACTCCTTCCA | CGGAATGCGATGCGTAGGAGTAGGAA | TTGTCCTAATGATGCTGGTCGCCCCATCCTACGGAATGCGATGCGTAGGAGTAGGAAACAGAGACTTTGTGGAAGGAGTCTCAGGTGGA |
| Kim_1_s | NC_001477.1 | GGTTGTAGGAGATGTTGCTGGG | TAGCCTTTCCCCAGCTTTTCCA | TGATTAGGCCACAACCCATGGAATACAA | GGTCGTAGGAGACGTTAGTGGAATCTTGGCCCAAGGAAAGAAAATGATTAGGCCACAACCCATGGAACACAAATACTCGTGGAAAAGCTGGGGAAAAGCCA |
| Kim_2_s | NC_001474.2 | TCAAAGGAATCATGCAGGCAGG | TCGGGGCCATCAATGAGAAA | TGCGGCCTCAGCCCACTGAGCTAAAGTA | TCAAAGGAATCATGCAGGCAGGAAAACGATCTCTGCGGCCTCAGCCCACTGAGCTGAAGTATTCATGGAAAACATGGGGCAAAGCAAAAATGCTCTCTACAGAGTCTCATAACCAGACCTTTCTCATTGATGGCCCCGA |
| Kim_3_s | NC_001475.2 | GCCAATGAACTGAACTACATATTATGGG | GGTGTTAGTGTTCTTTTCCCTTGCTCT | ACGGTAGTTGTGGGCGACATAATTGG | GCCAATGAACTGAACTACATATTATGGGAAAACAATATCAAATTAACGGTAGTTGTGGGCGATACACTTGGGGTCTTAGAGCAAGGGAAAAGAACACTAACACC |
| Kim_4_s | NC_002640.1 | ATGTTCTCTGGGAAGGAGGACAT | TCATTCACTGGAGGTGTGAGTGCTC | TCACTGTAGTGGCTGGGGACGTGAA | ATGTTCTCTGGGAAGGAGGACATGACCTCACTGTAGTGGCTGGGGATGTGAAGGGGGTGTTGACCAAAGGCAAGAGAGCACTCACACCCCCAGTGAGTGA |
| Kong_1_s | NC_001477.1 | GGAAGGAGAAGGACTGCACA | ATTCTTGTGTCCCATCCTGCT | CTCAGAGACATATCAAAGATTCCCGGG | GGAAGGAGAAGGACTCCACAAACTTGGATACATACTCAGAGACATATCAAAGATTCCAGGGGGAAATATGTATGCAGATGACACAGCCGGATGGGACACAAGAAT |
| Kong_2_s | NC_001474.2 | GGAAGGAGAAGGACTGCACA | ATTCTTGTGTCCCATCCTGCT | TAAGAGACGTGAGCAAGAAAGAGGGAGGAG | GGAAGGAGAAGGGCTGCACAAGCTAGGTTACATTCTAAGAGACGTGAGCAAGAAAGAGGGAGGAGCAATGTATGCCGATGACACCGCAGGATGGGATACAAGAAT |
| Kong_3_s | NC_001475.2 | GGAAGGAGAAGGACTGCACA | ATTCTTGTGTCCCATCCTGCT | ACATTTCCAAGATACCCGGAGGAG | AGAAGGAGAAGGACTGCACAAGCTAGGCTACATATTAAGGGACATTTCCAAGATACCCGGAGGAGCCATGTATGCTGATGACACAGCTGGTTGGGACACAAGAAT |
| Kong_4_s | NC_002640.1 | GGAAGGAGAAGGACTGCACA | ATTCTTGTGTCCCATCCTGCT | CCTAGAGGACATAGACAAAAAGGAAGGAGACC | GGAAGGGGAAGGTCTGCACAGATTGGGATATATCCTGGAGGAGATAGACAAGAAGGATGGAGACCTAATGTATGCTGATGACACAGCAGGCTGGGACACAAGAAT |
| Laue_1_s | NC_001477.2 | ATCCATGCCCATCACCAATG | CAGGGATCCACACCACTGATC | GACAAAACCCATGTATCCAGTTGGGAAGATGTT | ATCCATGCCCACCATCAATGGATGACAACAGAAGACATGTTGTCAGTGTGGAATAGGGTTTGGATAGAGGAAAACCCATGGATGGAGGACAAGACTCATGTGTCCAGTTGGGAAGACGTTCCATACCTAGGAAAAAGGGAAGATCAATGGTGTGGTTCCCTA |
| Laue_2_s | NC_001474.3 | ACAAGTCGAACAACCTGGTCCAT | GCCGCACCATTGGTCTTCTC | CCAGTGGAATCATGGGAGGAAATCCCA | ACAAGTCGAACAACCTGGTCCATACATGCTAAACATGAATGGATGACAACGGAAGACATGCTGACAGTCTGGAACAGGGTGTGGATTCAAGAAAACCCATGGATGGAAGACAAAACTCCAGTGGAATCATGGGAGGAAATCCCATACTTGGGGAAAAGAGAAGACCAATGGTGCGGC |
| Laue_3_s | NC_001475.3 | TGGCAACAGGTCCCTTTCTG | TGGCGTTGGATGCTAGTCTAAGA | AAGAAAGTTGGTTAGTTCCCTGCAGACCCCA | TGGCAACAGGTTCCTTTCTGCTCCCACCACTTTCATGAATTGATCATGAAAGATGGAAGAAAGTTGGTGGTTCCCTGCAGACCCCAGGACGAACTAATAGGAAGAGCAAGAATCTCTCAAGGAGCGGGATGGAGCCTTAGAGAAACTGCATGTCTGGGGAAAGCCTACGCCCAAATGTGGAGTCTCATGTATTTTCACAGAAGAGATCTCAGATTAGCATCCAACGCCA |
| Laue_4_s | NC_002640.2 | GCGTGGTGAAGCCCCTAGAT | GAACAACTAGTGAGCGGCCATC | TGGCACTTCCCTCCTCTTCTTGAACGAC | GCGTGGTGAAGCCCCTAGATGAGAGGTTTGGCACTTCCCTCCTCTTCTTGAACGACATGGGAAAGGTGAGGAAAGACATTCCGCAGTGGGAACCATCTAAGGGATGGAAAAACTGGCAAGAGGTTCCTTTTTGCTCCCACCACTTTCACAAGATCTTTATGAAGGATGGCCGCTCACTAGTTGTTC |
| Leparc_Goffart_1_g | NC_001477.1 | AGGACYAGAGGTTAGAGGAGA | CGYTCTGTGCCTGGAWTGAT | ACAGCATATTGACGCTGGGARAGACC | AGGACTAGAGGTTAGAGGAGACCCCCCGCACAACAACAAACAGCATATTGACGCTGGGAGAGACCAGAGATCCTGCTGTCTCTACAGCATCATTCCAGGCACAGAACG |
| Leparc_Goffart_2_g | NC_001474.2 | AGGACYAGAGGTTAGAGGAGA | CGYTCTGTGCCTGGAWTGAT | ACAGCATATTGACGCTGGGARAGACC | AGGACTAGAGGTTAGAGGAGACCCCCCCGAAACAAAAAACAGCATATTGACGCTGGGAAAGACCAGAGATCCTGCTGTCTCCTCAGCATCATTCCAGGCACAGAACG |
| Leparc_Goffart_3_g | NC_001475.2 | AGGACYAGAGGTTAGAGGAGA | CGYTCTGTGCCTGGAWTGAT | ACAGCATATTGACGCTGGGARAGACC | AGGACTAGAGGTTAGAGGAGACCCCCCGCAAATAAAAACAGCATATTGACGCTGGGAGAGACCAGAGATCCTGCTGTCTCCTCAGCATCATTCCAGGCACAGAACG |
| Leparc_Goffart_4_g | NC_002640.1 | AGGACYAGAGGTTAGAGGAGA | CGYTCTGTGCCTGGAWTGAT | ACAGCATATTGACGCTGGGARAGACC | AGGACTAGAGGTTAGAGGAGACCCCCCCAACACAAAAACAGCATATTGACGCTGGGAAAGACCAGAGATCCTGCTGTCTCTGCAACATCAATCCAGGCACAGAGCG |
| Leparc_Goffart_1_s | NC_001477.1 | ATACCYCCAACAGCAGGAATT | AGCATRAGGAGCATGGTCAC | TTGGCTAGATGGRGCTCATTCAAGAAGAAT | ATACCTCCAACAGCAGGAATTTTGGCTAGATGGGGCTCATTCAAGAAGAATGGAGCGATCAAAGTGTTACGGGGTTTCAAGAAAGAAATCTCAAACATGTTGAACATAATGAACAGGAGGAAAAGATCTGTGACCATGCTCCTCATGCT |
| Leparc_Goffart_2_s | NC_001474.2 | TGGACCGACAAAGACAGATTCTT | CGYCCYTGCAGCATTCCAA | CGCGAGAGAAACCGCGTGTCRACTGT | TGGACCGACAAAGACAGATTCTTTGAGGGAGCTAAGCTCAACGTAGTTCTAACAGTTTTTTAATTAGAGAGCAGATCTCTGATGAATAACCAACGGAAAAAGGCGAAAAACACGCCTTTCAATATGCTGAAACGCGAGAGAAACCGCGTGTCGACTGTGCAACAGCTGACAAAGAGATTCTCACTTGGAATGCTGCAGGGACG |
| Leparc_Goffart_3_s | NC_001475.2 | AAGACGGGAAAACCGTCTATCAA | TTGAGAATCTCTTCGCCAACTG | ATGCTGAAACGCGTGAGAAACCGTGT | AAGACGGGAAAACCGTCTATCAATATGCTGAAACGCGTGAGAAACCGTGTGTCAACTGGATCACAGTTGGCGAAGAGATTCTCAA |
| Leparc_Goffart_4_s | NC_002640.1 | CCATCCCACCRACAGCAGG | CAAGATGTTCAGCATGCGGC | ATGGGGACAGTTRAAGAAAAAYAAGGCCAT | CCATCCCACCAACAGCAGGGATTCTGAAGAGATGGGGACAGTTGAAGAAAAATAAGGCCATCAAGATACTGATTGGATTCAGGAAGGAGATAGGCCGCATGCTGAACATCTTG |
| Pongsiri_1_g | NC_001477.1 | GACTAGYGGTTAGAGGAGACC | GHRAGACAGCAGGATCTCTG | AAGGACTAGMGGTTAGWGGAGACCC | GACTAGTGGTTAGAGGAGACCCCTCCCAAGACACAACGCAGCAGCGGGGCCCAACACCAGGGGAAGCTGTACCCTGGTGGTAAGGACTAGAGGTTAGAGGAGACCCCCCGCACAACAACAAACAGCATATTGACGCTGGGAGAGACCAGAGATCCTGCTGTCTCTA |
| Pongsiri_2_g | NC_001474.2 | GACTAGYGGTTAGAGGAGACC | GHRAGACAGCAGGATCTCTG | AAGGACTAGMGGTTAGWGGAGACCC | GACTAGCGGTTAGAGGAGACCCCTCCCTTACAAATCGCAGCAACAATGGGGGCCCAAGGCGAGATGAAGCTGTAGTCTCGCTGGAAGGACTAGAGGTTAGAGGAGACCCCCCCGAAACAAAAAACAGCATATTGACGCTGGGAAAGACCAGAGATCCTGCTGTCTCCT |
| Pongsiri_3_g | NC_001475.2 | GACTAGYGGTTAGAGGAGACC | GHRAGACAGCAGGATCTCTG | AAGGACTAGMGGTTAGWGGAGACCC | GACTAGCGGTTAGAGGAGACCCCTCCCATGACACAACGCAGCAGCGGGGCCCGAGCACTGAGGGAAGCTGTACCTCCTTGCAAAGGACTAGAGGTTAGAGGAGACCCCCCGCAAATAAAAACAGCATATTGACGCTGGGAGAGACCAGAGATCCTGCTGTCTCCT |
| Pongsiri_4_g | NC_002640.1 | GACTAGYGGTTAGAGGAGACC | GHRAGACAGCAGGATCTCTG | AAGGACTAGMGGTTAGWGGAGACCC | GACTAGCGGTTAGAGGAGACCCCTCCCATCACTGATAAAACGCAGCAAAAGGGGGCCCGAAGCCAGGAGGAAGCTGTACTCCTGGTGGAAGGACTAGAGGTTAGAGGAGACCCCCCCAACACAAAAACAGCATATTGACGCTGGGAAAGACCAGAGATCCTGCTGTCTCTG |
| Sadon_1_s | NC_001477.1 | TGATGAACAACCAACGRAAAAA | GTTTCTCCCGCGTTTCAGCAT | CGGSTCGACCGTCTTTC | TGATGAACAACCAACGGAAAAAGACGGGTCGACCGTCTTTCAATATGCTGAAACGCGCGAGAAAC |
| Sadon_2_s | NC_001474.2 | CTGCARGGACGAGGACCATT | GGGATTGTTAGGAAACGAAGGA | AAACTGTTCATGGCCCTGGTGGCR | CTGCAGGGACGAGGACCATTAAAACTGTTCATGGCCCTGGTGGCGTTCCTTCGTTTCCTAACAATCCC |
| Sadon_3_s | NC_001475.2 | TCGCTCTGTCTCATGATGATRTT | GGCTCTCCATCGCGTGAA | CCAGCAACACTTGCTTTCCACTT | TCGCTCTGTCTCATGATGATATTGCCAGCAGCACTTGCTTTCCACTTGACTTCACGAGATGGAGAGCC |
| Sadon_4_s | NC_002640.1 | TCTCTGGAAAAATGAACCAACGA | CGGTTTCTCTCGCGTTTCAG | AAAAGGTGGTTAGACCACCTTTCAATAT | TCTCTGGAAAAATGAACCAACGAAAAAAGGTGGTTAGACCACCTTTCAATATGCTGAAACGCGAGAGAAACCG |
| Santiago_1_s | NC_001477.1 | CAAAAGGAAGTCGYGCAATA | CTGAGTGAATTCTCTCTGCTRAAC | CATGTGGYTGGGAGCRCGC | CAAAAGGAAGTCGCGCAATATGGTACATGTGGTTGGGAGCGCGCTTTTTAGAGTTTGAAGCCCTTGGTTTCATGAATGAAGATCACTGGTTCAGCAGAGAGAATTCACTCAG |
| Santiago_2_s | NC_001474.2 | CAGGCTATGGCACYGTCACGAT | CCATYTGCAGCARCACCATCTC | CTCYCCRAGAACGGGCCTCGACTTCAA | CAGGTTATGGCACTGTCACAATGGAGTGCTCTCCAAGAACGGGCCTCGACTTCAATGAGATGGTGTTGCTGCAGATGG |
| Santiago_3_s | NC_001475.2 | GGACTRGACACACGCACCCA | CATGTCTCTACCTTCTCGACTTGYCT | ACCTGGATGTCGGCTGAAGGAGCTTG | GGACTGGACACACGCACCCAAACCTGGATGTCGGCTGAAGGAGCTTGGAGACAAGTCGAGAAGGTAGAGACATG |
| Santiago_4_s | NC_002640.1 | TTGTCCTAATGATGCTRGTCG | TCCACCYGAGACTCCTTCCA | CGGAATGCGATGCGTAGGRGTAGGRA | TTGTCCTAATGATGCTGGTCGCCCCATCCTACGGAATGCGATGCGTAGGAGTAGGAAACAGAGACTTTGTGGAAGGAGTCTCAGGTGGA |
| Warrilow_1_g | NC_001477.1 | AAGGACTAGAGGTTAKAGGAGACCC | CGWTCTGTGCCTGGAWTGATG | AACAGCATATTGACGCTGGGAAAGACCAGA | AAGGACTAGAGGTTAGAGGAGACCCCCCGCACAACAACAAACAGCATATTGACGCTGGGAGAGACCAGAGATCCTGCTGTCTCTACAGCATCATTCCAGGCACAGAACG |
| Warrilow_2_g | NC_001474.2 | AAGGACTAGAGGTTAKAGGAGACCC | CGWTCTGTGCCTGGAWTGATG | AACAGCATATTGACGCTGGGAAAGACCAGA | AAGGACTAGAGGTTAGAGGAGACCCCCCCGAAACAAAAAACAGCATATTGACGCTGGGAAAGACCAGAGATCCTGCTGTCTCCTCAGCATCATTCCAGGCACAGAACG |
| Warrilow_3_g | NC_001475.2 | AAGGACTAGAGGTTAKAGGAGACCC | CGWTCTGTGCCTGGAWTGATG | AACAGCATATTGACGCTGGGAAAGACCAGA | AAGGACTAGAGGTTAGAGGAGACCCCCCGCAAATAAAAACAGCATATTGACGCTGGGAGAGACCAGAGATCCTGCTGTCTCCTCAGCATCATTCCAGGCACAGAACG |
| Warrilow_4_g | NC_002640.1 | AAGGACTAGAGGTTAKAGGAGACCC | CGWTCTGTGCCTGGAWTGATG | AACAGCATATTGACGCTGGGAAAGACCAGA | AGGACTAGAGGTTAGAGGAGACCCCCCCAACACAAAAACAGCATATTGACGCTGGGAAAGACCAGAGATCCTGCTGTCTCTGCAACATCAATCCAGGCACAGAGCG |

**Table legend:** The first column lists the method name (see Table 1, Methods). The second column lists the NCBI accession number of the reference genome used to extract the template reference for the targeted genomic region. The third, fourth, and fifth columns provide the sequence information for the forward primer, reverse primer and probe, respectively, which was retrieved for each method from their corresponding reference publication (see Table 1). The sixth column lists the sequence information for the reference template for the targeted genomic region, and was obtained through manually blasting the primer and probe sequences to the reference genome followed by manual curation. All sequences are listed in a 5’ to 3’ orientation.

## Supporting data: Input and output data employed and generated in this study

### Input data

The associated file ‘inputSCREENED.zip’ contains all data employed as input for this study:

- File ‘dengue1.fasta’: Genomic sequences employed for dengue virus serotype 1
- File ‘dengue2.fasta’: Genomic sequences employed for dengue virus serotype 2
- File ‘dengue3.fasta’: Genomic sequences employed for dengue virus serotype 3
- File ‘dengue4.fasta’: Genomic sequences employed for dengue virus serotype 4
- File ‘westnileVirus.fasta’: Genomic sequences employed for West Nile virus
- File ‘DengueConfigFile.txt’: Configuration file that contains all sequence information for the forward and reverse primers, the probe, and the reference template for the targeted genomic region, for every RT-qPCR method. This corresponds to Supplementary Table S1 in a tab-delimited format that can be employed to run SCREENED.

### Output data

The associated file ‘outputSCREENED.zip’ contains all data generated as output for this study subdivided in six folders:

- Folder ‘sensitivity_dengue_mismatch20_length80_end5_bases0’: Contains all output of SCREENED when evaluating the sensitivity of all dengue virus RT-qPCR methods (see Table 1), using a maximum of 20% allowed mismatches, a minimum alignment length of 80%, and no single mismatch allowed in the last five bases at the 3’ end for the forward and reverse primers.
- Folder ‘sensitivity_dengue_mismatch20_length80_end5_bases1’: Contains all output of SCREENED when evaluating the sensitivity of all dengue virus RT-qPCR methods (see Table 1), using a maximum of 20% allowed mismatches, a minimum alignment length of 80%, and one mismatch allowed in the last five bases at the 3’ end for the forward and reverse primers.
- Folder ‘specificity_dengue_mismatch20_length80_end5_bases0’: Contains all output of SCREENED when evaluating the intraspecies specificity of all dengue virus serotype-specific RT-qPCR methods (see Table 1), using a maximum of 20% allowed mismatches, a minimum alignment length of 80%, and no single mismatch allowed in the last five bases at the 3’ end for the forward and reverse primers.
- Folder ‘specificity_dengue_mismatch20_length80_end5_bases1’: Contains all output of SCREENED when evaluating the intraspecies specificity of all dengue virus serotype-specific RT-qPCR methods (see Table 1), using a maximum of 20% allowed mismatches, a minimum alignment length of 80%, and one mismatch allowed in the last five bases at the 3’ end for the forward and reverse primers.
- Folder ‘specificity_westNile_mismatch20_length80_end5_bases0’: Contains all output of SCREENED when evaluating the interspecies specificity of all dengue virus RT-qPCR methods (see Table 1), using a maximum of 20% allowed mismatches, a minimum alignment length of 80%, and no single mismatch allowed in the last five bases at the 3’ end for the forward and reverse primers.
- Folder ‘specificity_westNile_mismatch20_length80_end5_bases1’: Contains all output of SCREENED when evaluating the interspecies specificity of all dengue virus RT-qPCR methods (see Table 1), using a maximum of 20% allowed mismatches, a minimum alignment length of 80%, and one mismatch allowed in the last five bases at the 3’ end for the forward and reverse primers.

## Supporting information: Extension of local alignments retrieved by BLAST

Because BLASTN is based on a local alignment strategy, it was sometimes observed that the algorithm could not recover the entire targeted genomic region at its ends due to mismatches in the first or last few bases. For such particular cases, the sequence of the genomic region was extended based on the specific genomic sequence under investigation. An illustratory example is provided below. For the genome with serotype 1 and NCBI accession HM181954 (see associated file ‘inputSCREENED.zip’), when searching for the targeted genomic region using the template reference for the method ‘Kong_1_s’ (see Supplementary Table S1), the following alignment was produced by BLAST:


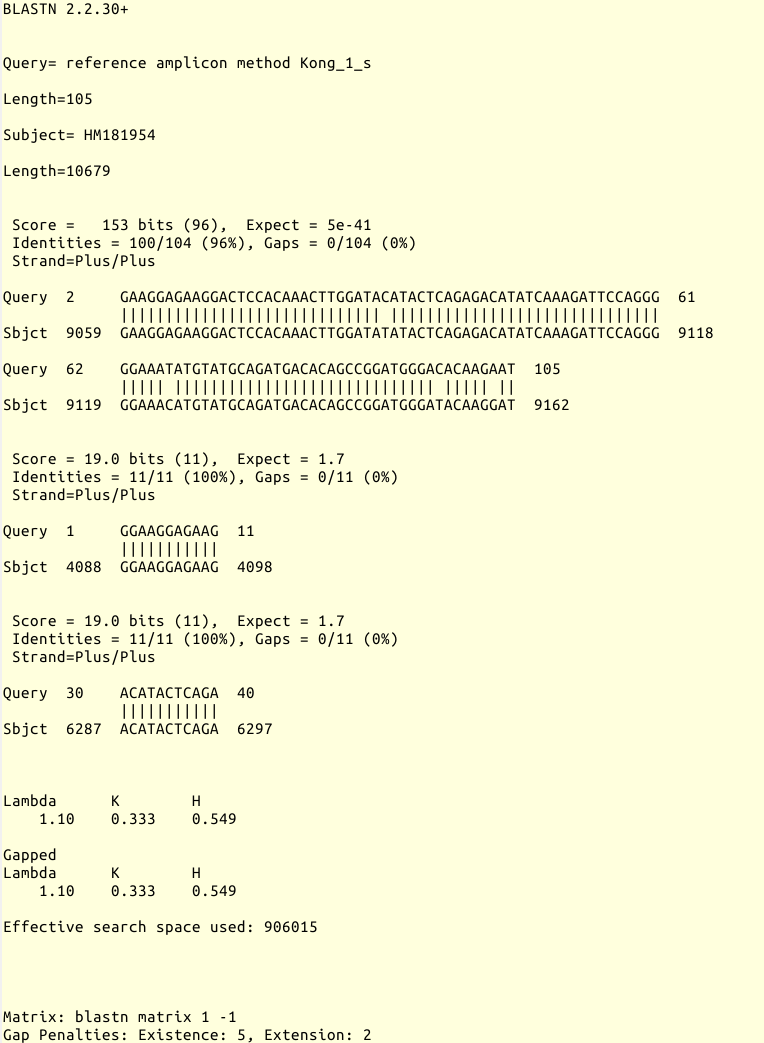


A hit is found within the subject genome over the entire length of the template reference for the targeted region (105 bases), with the exception of the first nucleotide at its 5’ end, since the BLAST alignment starts at position two of the query. The sequences for the template reference and recovered genomic region for this example are therefore ‘GGAAGGAGAAGGACTCCACAAACTTGGATACATACTCAGAGACATATCAAAGATTCCAGGGGGAAATATGTATGCAGATGACACAGCCGGATGGGACACAAGAAT’ and ‘GAAGGAGAAGGACTCCACAAACTTGGATATATACTCAGAGACATATCAAAGATTCCAGGGGGAAACATGTATGCAGATGACACAGCCGGATGGGATACAAGGAT’, respectively. The first base is missing in the recovered genomic region due to the fact that the genomic subject contains the base ‘A’ at position 9058, whereas the template reference contains the base ‘G’ at its first position. Since the BLAST algorithm cannot overcome such mismatches at the end of a local alignment, this base is missing incorrectly from the recovered genomic region. For such cases, an automated extension step was therefore performed by extending the local alignment with a number of bases that is determined by the length of the reference template. Added bases were always extracted based on their corresponding position in the analysed genome (i.e. the base ‘A’ was added at the 5’ end of the recovered genomic region sequence for this example), resulting in the sequence ‘AGAAGGAGAAGGACTCCACAAACTTGGATATATACTCAGAGACATATCAAAGATTCCAGGGGGAAACATGTATGCAGATGACACAGCCGGATGGGATACAAGGAT’. Extension was never performed past the original length of the reference template.

Similarly, it was sometimes observed that the algorithm could not recover the entire targeted annealing site at its ends due to mismatches in the first or last few bases. For such particular cases, the sequence of the targeted annealing site was extended based on the specific genomic sequence under investigation. An illustratory example is provided below. For the genome with serotype 2 and NCBI accession KX380822 (see associated file ‘inputSCREENED.zip’), the following recovered genomic region was obtained for the method ‘Conceicao_2_g’: ‘TTTAGAGAGCAGATCTCTGATGAATAACCAACGGAAAAAGGCGAGAAATACGCCTTTCAATATGCTGAAACGCGAGAGAAACCGCGTGTCA’. When searching within this sequence for the forward primer (see Supplementary Table S1), the following alignment was produced by BLAST:


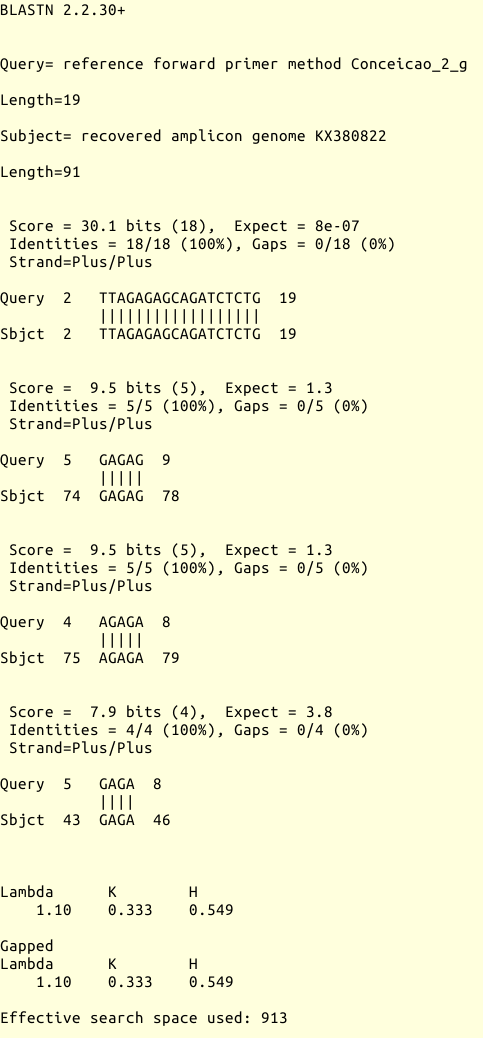


A hit is found within the subject recovered genomic region over the entire length of the query forward primer (19 bases), with the exception of the first nucleotide at its 5’ end, since the BLAST alignment starts at position two of the query. The sequences for the forward primer and its recovered annealing site for this example are therefore ‘ATTAGAGAGCAGATCTCTG’ and ‘TTAGAGAGCAGATCTCTG’, respectively. Investigation indicates that the first base is missing in the recovered forward primer annealing site due to the fact that the recovered genomic region subject contains the base ‘T’ at position 1, whereas the forward primer sequence contains the base ‘A’ at its first position. Since the BLAST algorithm cannot overcome such mismatches at the beginning of an alignment, this base is missing incorrectly from the recovered forward primer annealing site. For such cases, an automated extension step was therefore performed that extended the recovered annealing sequence with a number of bases that is determined by the length of the forward primer sequence. Added bases were always extracted based on their corresponding position in the recovered genomic region (i.e*.* the base ‘T’ was added at the 5’ end of the recovered forward primer annealing site for this example), resulting in the sequence ‘TTTAGAGAGCAGATCTCTG’. Extension was never performed past the original length of the primer sequence.

## Supporting information: Unknown cases most likely represent incomplete genomic sequences

Figure 1 (see main manuscript) illustrates how our approach distinguishes two cases of unknown outcomes. First, genomes where no single BLAST hit for the targeted genomic region could be retrieved were considered as unknown because this could either indicate that the genomic sequence is incomplete, or alternatively that the genome in reality does not contain the targeted genomic region. Second, genomes where a targeted genomic region has been retrieved that is located at the boundary of the genomic sequence but is not detected, were also considered as unknown because this could either indicate that the genomic sequence is incomplete, or alternatively that the targeted genomic region is present but does not lead to detection. For the selection criteria employed for Table 2 of the main manuscript, this results in the following contingency table when one mismatched base is allowed at the 3’ end of primer annealing sites:

|  |  | **Outcome** | | |
| --- | --- | --- | --- | --- |
|  |  | **Unknown** | **Other** | **Row total** |
| **RT-qPCR method** | **Non-UTR method** | 690 | 30648 | 31338 |
|  | **UTR method** | 2087 | 25951 | 28038 |
|  | **Column total** | 2777 | 56599 | 59376 |

**Table SI1:** Contingency table illustrating the overrepresentation of unknown cases for RT-qPCR methods that do versus don’t target an UTR method, referred to as ‘UTR method’ and ‘non-UTR method’, respectively, for results presented in Table 2 when one mismatched base is allowed at the 3’ end of primer annealing sites. ‘Unknown’ and ‘other’ refer to genomes classified as unknown, or (not) detected, respectively (see Figure 1).

A statistically significant overrepresentation (Fisher’s Exact Test, *p* < 1e-15) of unknown cases was observed for methods that do versus don’t target the UTR regions: 2,087 out of 28,038 cases (7.44%) versus 690 out of 31,338 cases (2.20%). For the selection criteria employed for Table 2 of the main manuscript when no single mismatched base is allowed at the 3’ end of primer annealing sites, this results in the following contingency table:

|  |  | **Outcome** | | |
| --- | --- | --- | --- | --- |
|  |  | **Unknown** | **Other** | **Row total** |
| **RT-qPCR method** | **Non-UTR method** | 690 | 30648 | 31338 |
|  | **UTR method** | 2119 | 25919 | 28038 |
|  | **Column total** | 2809 | 56567 | 59376 |

**Table SI2:** Contingency table illustrating the overrepresentation of unknown cases for RT-qPCR methods that do versus don’t target an UTR method, referred to as ‘UTR method’ and ‘non-UTR method’, respectively, for results presented in Table 2 when no single mismatched base is allowed at the 3’ end of primer annealing sites. ‘Unknown’ and ‘other’ refer to genomes classified as unknown, or (not) detected, respectively (see Figure 1).

A statistically significant overrepresentation (Fisher’s Exact Test, *p* < 1e-15) of unknown cases was again observed for methods that do versus don’t target the UTR regions: 2,119 out of 28,038 cases (7.56%) versus 690 out of 31,338 cases (2.20%).

Furthermore, upon closer inspection, 688 out of 690 unknown cases for non-UTR methods derive from one particular method, namely ‘Leparc_Goffart_2_s’, which targets the beginning of the capsid region that is located immediately after the end of the 5’UTR region [1]. For all 688 cases, this always corresponded with the first base of the available genomic sequence. Grouping this method with UTR methods as targeting the genomic extremities renders the above trend even more evident for methods that do versus don’t target the extremities. For the selection criteria employed for Table 2 of the main manuscript, this results in the following contingency table when one mismatched base is allowed at the 3’ end of primer annealing sites:

|  |  | **Outcome** | | |
| --- | --- | --- | --- | --- |
|  |  | **Unknown** | **Other** | **Row total** |
| **RT-qPCR method** | **Non- genomic extremity method** | 2 | 30172 | 30174 |
|  | **Genomic extremity method** | 2775 | 26427 | 29202 |
|  | **Column total** | 2777 | 56599 | 59376 |

**Table SI3:** Contingency table illustrating the overrepresentation of unknown cases for RT-qPCR methods that do versus don’t target a genomic extremity, referred to as ‘genomic extremity method’ and ‘non-genomic extremity method’, respectively, for results presented in Table 2 when one mismatched base is allowed at the 3’ end of primer annealing sites. ‘Unknown’ and ‘other’ refer to genomes classified as unknown, or (not) detected, respectively (see Figure 1).

A statistically significant overrepresentation (Fisher’s Exact Test, *p* < 1e-15) of unknown cases was observed for methods that do versus don’t target the genomic extremities: 2,775 out of 29,202 cases (9.50%) versus 2 out of 30,174 cases (<0.01%). For the selection criteria employed for Table 2 of the main manuscript when no single mismatched base is allowed at the 3’ end of primer annealing sites, this results in the following contingency table:

|  |  | **Outcome** | | |
| --- | --- | --- | --- | --- |
|  |  | **Unknown** | **Other** | **Row total** |
| **RT-qPCR method** | **Non-genomic extremity method** | 2 | 30172 | 30174 |
|  | **Genomic extremity method** | 2807 | 26395 | 29202 |
|  | **Column total** | 2809 | 56567 | 59376 |

**Table SI4:** Contingency table illustrating the overrepresentation of unknown cases for RT-qPCR methods that do versus don’t target a genomic extremity, referred to as ‘genomic extremity method’ and ‘non-genomic extremity method’, respectively, for results presented in Table 2 when no single mismatched base is allowed at the 3’ end of primer annealing sites. ‘Unknown’ and ‘other’ refer to genomes classified as unknown, or (not) detected, respectively (see Figure 1).

A statistically significant overrepresentation (Fisher’s Exact Test, *p* < 1e-15) of unknown cases was again observed for methods that do versus don’t target the genomic extremities: 2,807 out of 29,202 cases (9.61%) versus 2 out of 30174 cases (<0.01%).

Combined, these observations support that unknown cases most likely are caused by incomplete genomic sequence information for the analysed genomes at their boundaries, supporting that such observations should not be included in the calculation of sensitivities for each RT-qPCR method.

## Supporting information: The low *in silico* performance of the method developed by Chien et al. can be explained by primer mismatches in the last five bases at their 3’ end

Table 2 (see main manuscript) indicates that the method developed by Chien et al. [2] for serotype-specific detection performs relatively poorly (6.95% *in silico* sensitivity), when no single mismatch is allowed in the last five bases at the 3’ end of the forward and reserve primers. A detailed investigation of these particular cases was performed to classify the mutations that lead to the method being rejected. Table SI5 lists the number of analysed genomes displaying specific mutations in the last five bases at the 3’ end of the forward and/or reverse primer(s) for serotypes 1, 2, and 3 (the fourth serotype attains a high score of 98.35% *in silico* sensitivity):

| **Chien_1_s** | | | **Chien_2_s** | | | **Chien_3_s** | | |
| --- | --- | --- | --- | --- | --- | --- | --- | --- |
| Primer | Mutation | # Genomes | Primer | Mutation | # Genomes | Primer | Mutation | # Genomes |
| Forward | A->G | 6/1359 | Forward | A->G | 1113/1164 | Forward | A->G | 716/777 |
| Reverse | T->G | 3/1359 | Reverse | T->C | 998/1164 | Reverse | T->C | 87/777 |
| Reverse | T->A | 1351/1359 | Reverse | T->A | 16/1164 | Reverse | T->A | 1/777 |

**Table SI5**: Mismatches in the last five bases at the 3’ end for the forward and reverse primers for the method developed by Chien et al. [2].

For serotype 1, thymine to adenine or guanine substitutions in the last five bases at the 3’ end occur in 1,354 genomes for the reverse primer, of which six also exhibit adenine to guanine substitutions in the last five bases at the 3’ end for the forward primer. For serotype 2, adenine to guanine substitutions in the last five bases at the 3’ end occur in 1,113 genomes for the forward primer, of which 964 also exhibit thymine to cytosine or adenine substitutions in the last five bases at the 3’ end for the reverse primer. Another 50 genomes exhibit similar thymine to cytosine or adenine substitutions in their reverse primer. For serotype 3, adenine to guanine substitutions in the last five bases at the 3’ end occur in 716 genomes for the forward primer, of which 85 also exhibit thymine to cytosine or adenine substitutions in the last five bases at the 3’ end for the reverse primer. This pattern was also observed in the reverse primer in three additional genomes. An optimized design of these primers, for instance by introducing nucleotide degeneracies at the aforementioned positions, could hence potentially increase method performance, although this would need to be validated in the wetlab first.

## Supporting information: Tutorial for using SCREENED

### Objective and purpose

The purpose of this tutorial is to get users acquainted with using SCREENED (‘polymeraSe Chain Reaction Evaluation through largE-scale miNing of gEnomic Data’). SCREENED has been made available as a tool using the Galaxy Workflow Management System [3], at Sciensano. Much more extensive tutorials about using Galaxy can be found at https://galaxyproject.org/learn, but this minimal user guide will demonstrate how the analyses presented in this study can be duplicated to get users started with their own specific RT-qPCR methods.

### Accessing Galaxy

Open your browser (Chrome or Firefox are recommended), and navigate to https://galaxy.sciensano.be. You will see the welcome screen where by clicking ‘Next’ you agree to read and to comply with the usage policy (see Figure SI1).


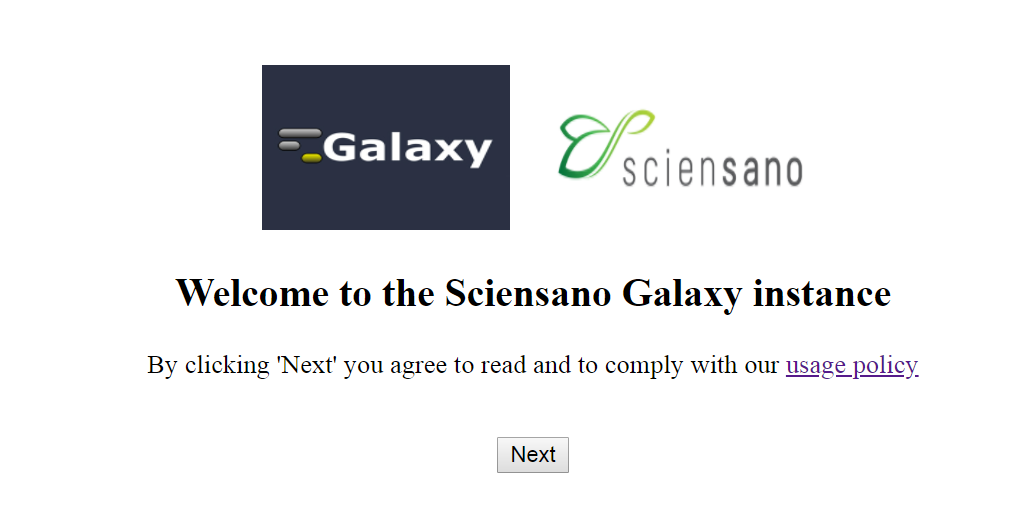


**Figure SI1:** The welcome screen for the Galaxy instance hosted by Sciensano.

You will be redirected to a page with the login screen (see Figure SI2). If you don’t have an account yet, you can create one by clicking ‘This Galaxy instance has been configured such that only users who are logged in may use it. If you don’t already have an account, you may create one’.


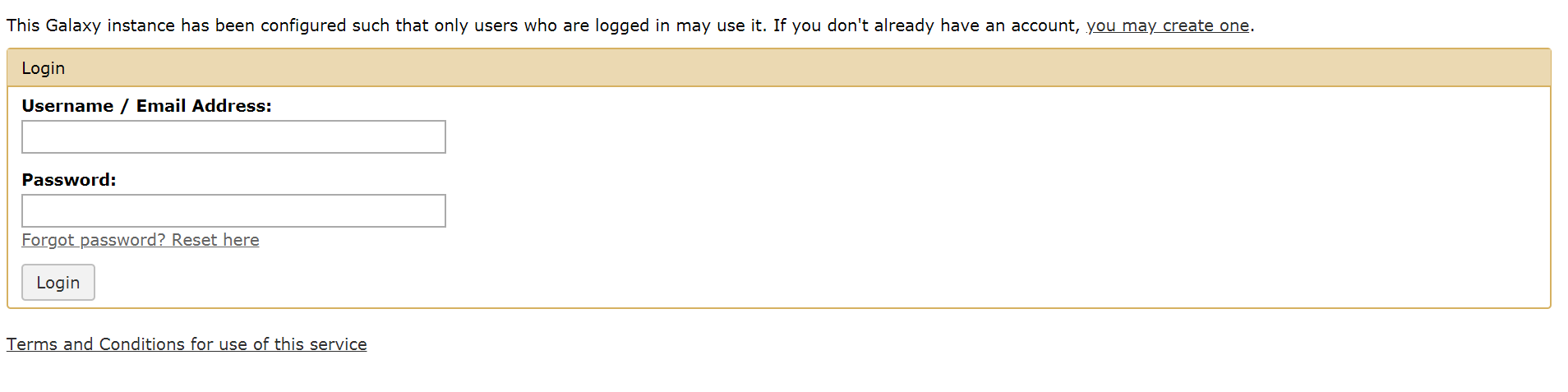


**Figure SI2:** The login screen for Galaxy where you can also create an account.

This will redirect you to the account creation screen, where you can create an account by providing an email address, a password and a public name (see Figure SI3). By clicking ‘Submit’, an account will be generated and you will be forwarded to the Galaxy instance.


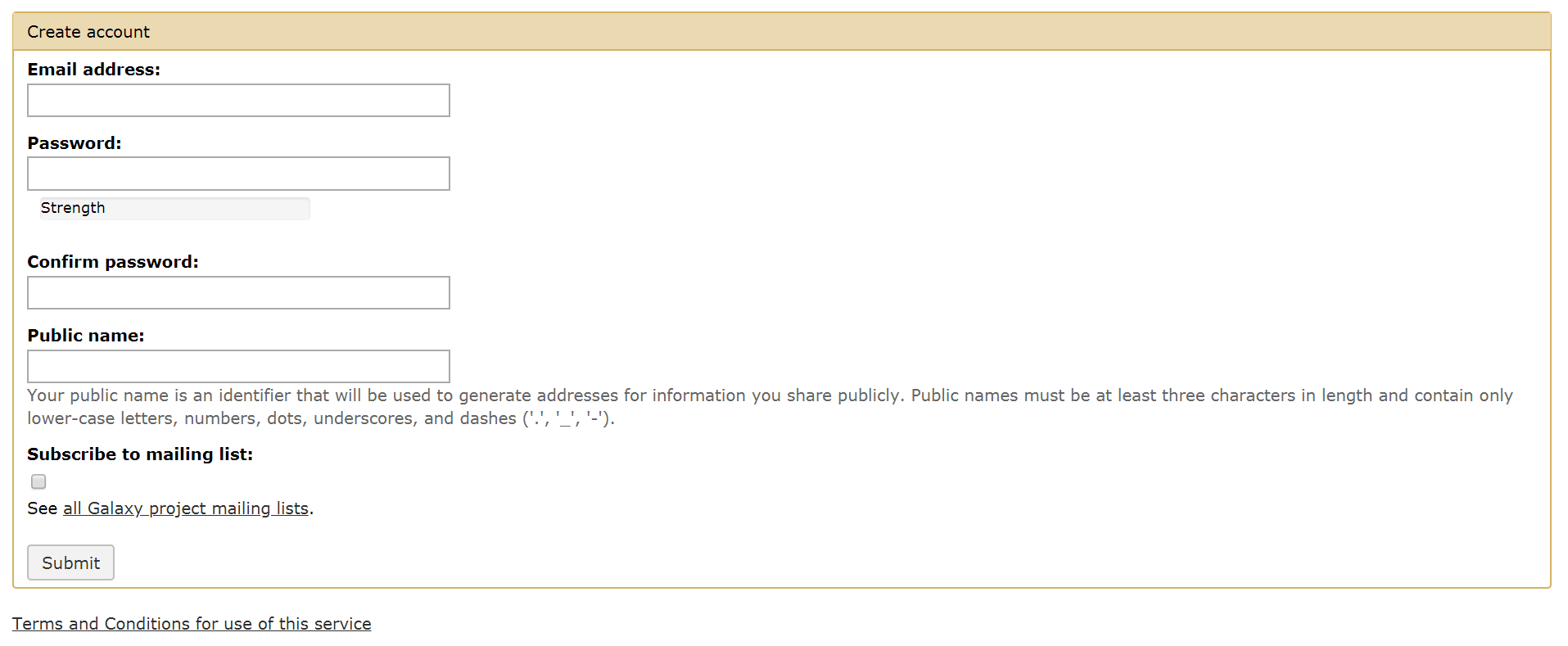


**Figure SI3:** The Galaxy account creation screen.

### Uploading data

Unzip the files contained within the associated file ‘inputSCREENED.zip’ (see Supporting Data) and place these files somewhere on your computer. Files can be uploaded into Galaxy by clicking ‘Get Data’ -> ‘Upload File’ in the Tools panel (see Figure SI4).


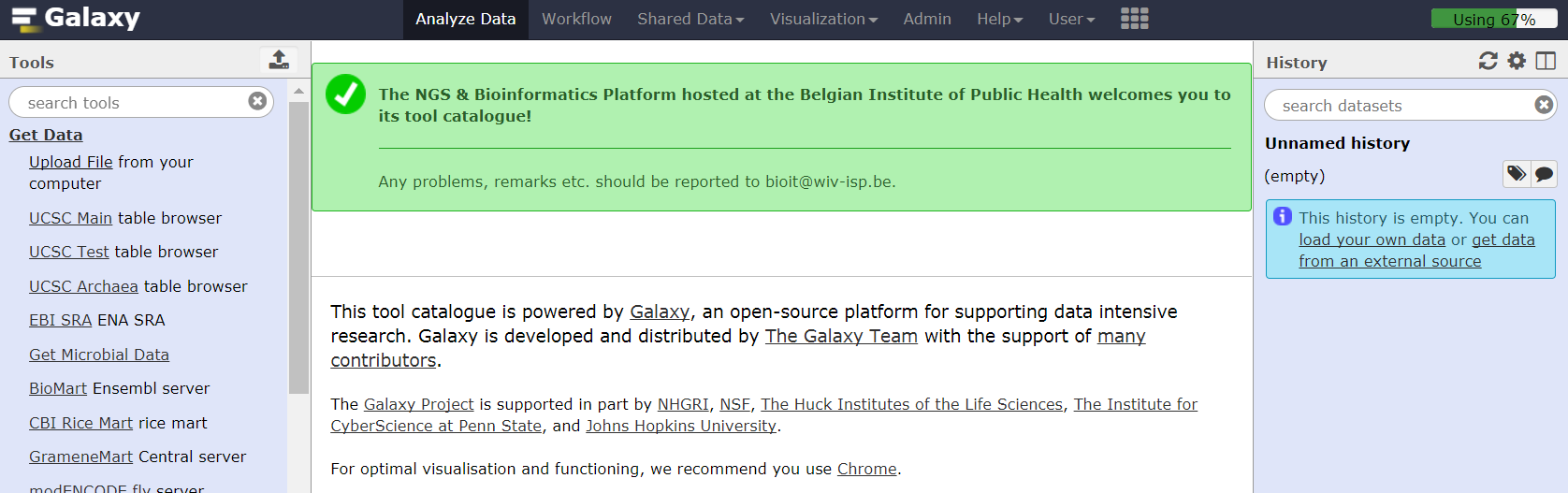


**Figure SI4:** Uploading files into Galaxy.

The upload interface will pop up. Upload the **unzipped files** from your computer by pressing ‘Choose local file’ (multiple files can be selected at once). Afterwards, set ‘Type’ to ‘fasta’ for the files ‘dengue1.fasta’, ‘dengue2.fasta’, ‘dengue3.fasta’, and ‘dengue4.fasta’. Set ‘Type’ to ‘tabular’ for the file ‘DengueConfigFile.txt’. Press ‘Start’ to commence upload of files (see Figure SI5). **Important: It is imperative to set the type of these files correctly. SCREENED will not run if fasta files do not receive the type ‘fasta’, and a config file with the type ‘tabular’.**


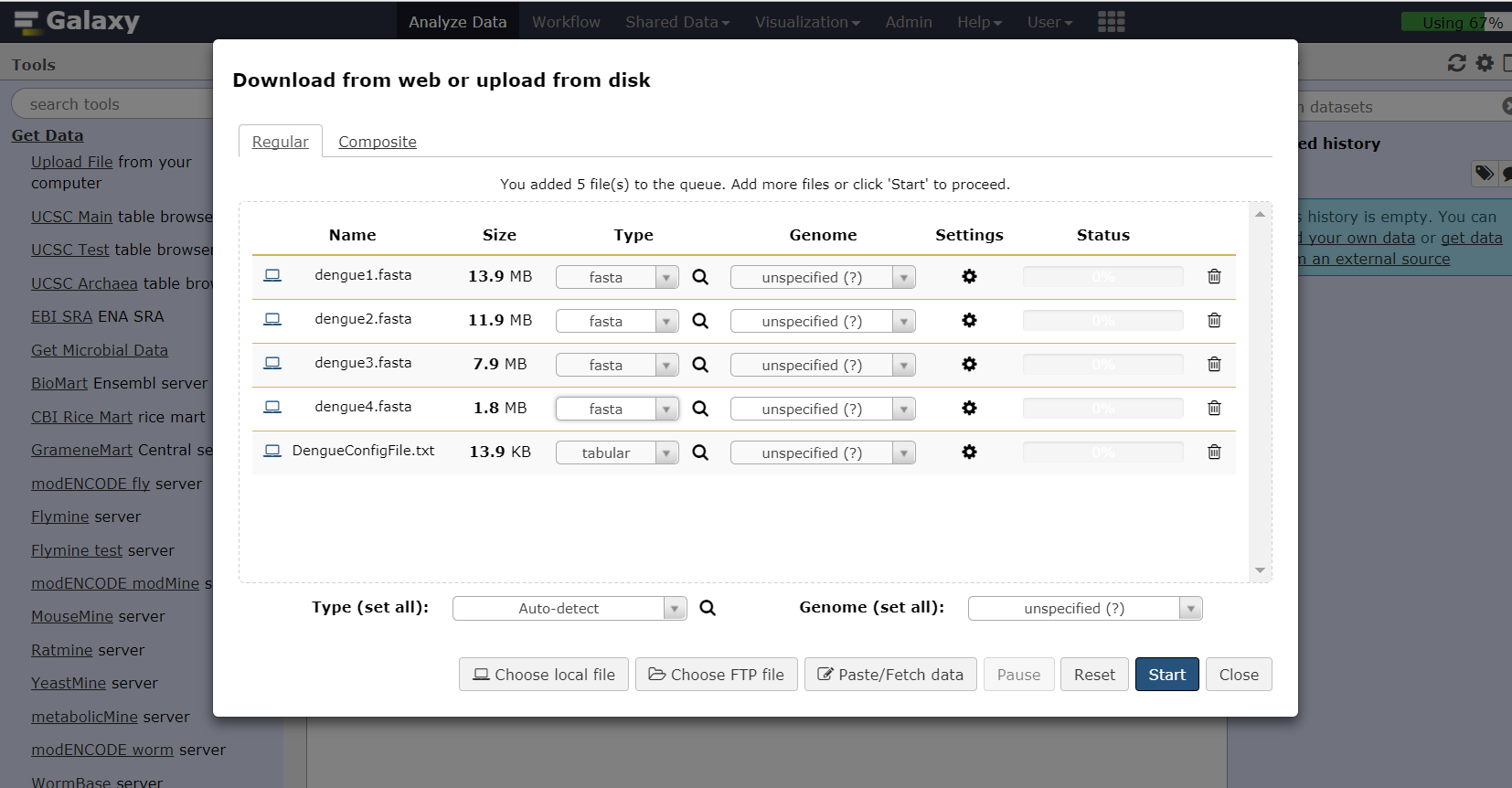


**Figure SI5:** The upload interface. Select local files by clicking ‘Choose local file’, and ensure afterwards that the ‘Type’ for every file is set correctly. Finish by clicking ‘Start’.

### Running SCREENED

When uploading has finished, the uploaded files will appear in the History panel on the right side. SCREENED can be accessed by clicking ‘SCREENED’ under the ‘SCREENED’ section in the Tools panel on the left. The tool interface will open in the middle panel (see Figure SI6).


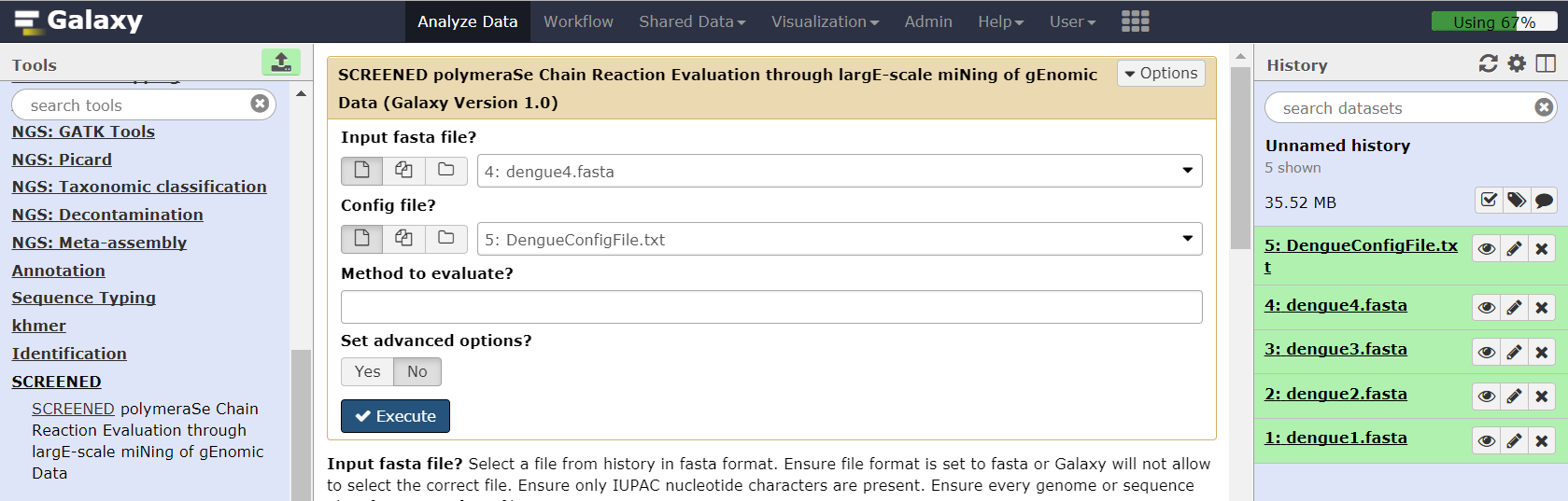


**Figure SI6:** SCREENED interface. Select SCREENED in the Tools panel on the left. Note that many different tools can be listed here (depending on which tools are currently installed within Galaxy), but SCREENED will always be available as its own category, or can be searched for by using ‘search tools’. Data are available in the History panel on the right. The middle panel will show the SCREENED interface. Note that the correct fasta file, and config file, need to be set based on the drop-down list. The ‘Method to evaluate?’ still needs to be set here to the method of interest, which needs to correspond with the listed methods in the file ‘DengueConfigFile.txt’ (case-sensitive).

By default, the last fasta file (‘4: dengue4.fasta’) will be automatically selected under ‘Input fasta file?’. Other fasta files can be selected by clicking the arrow next to this item. Select the file ‘1: dengue1.fasta’ to run SCREENED for the genomes available for serotype 1. By default, the correct config file will be selected under ‘Config file’. Under ‘Method to evaluate?’, type the name of the method to evaluate. For instance, to evaluate the method developed by Callahan et al. [4] for serotype-specific detection of the first serotype, type ‘Callahan_1_s’ (without the quotes), because this is how the method is named in the config file. **Important: It is imperative to provide a method name that corresponds with one of the method identifiers from the config file (case-sensitive). If either no method name is provided, or a method name that does not correspond exactly with a method identifier from the config file, SCREENED will fail to run!** Leave ‘Set advanced options?’ to ‘No’ to run the analysis using exactly the same settings as used in the main manuscript for Table 2, when one mismatched base is allowed at the 3’ end of primers. Finally, press ‘Execute’ to launch SCREENED. The middle panel will change to indicate that the tool has been successfully launched, and three new boxes containing the output will appear in the History panel. These boxes will be originally grey to indicate the jobs are queued, change to yellow to indicate the jobs are running, and finally turn green when the jobs are ready (see Figure SI7). If the boxes would turn red, the execution has failed. Review your settings in this case to ensure all options were set correctly and launch the tool again.


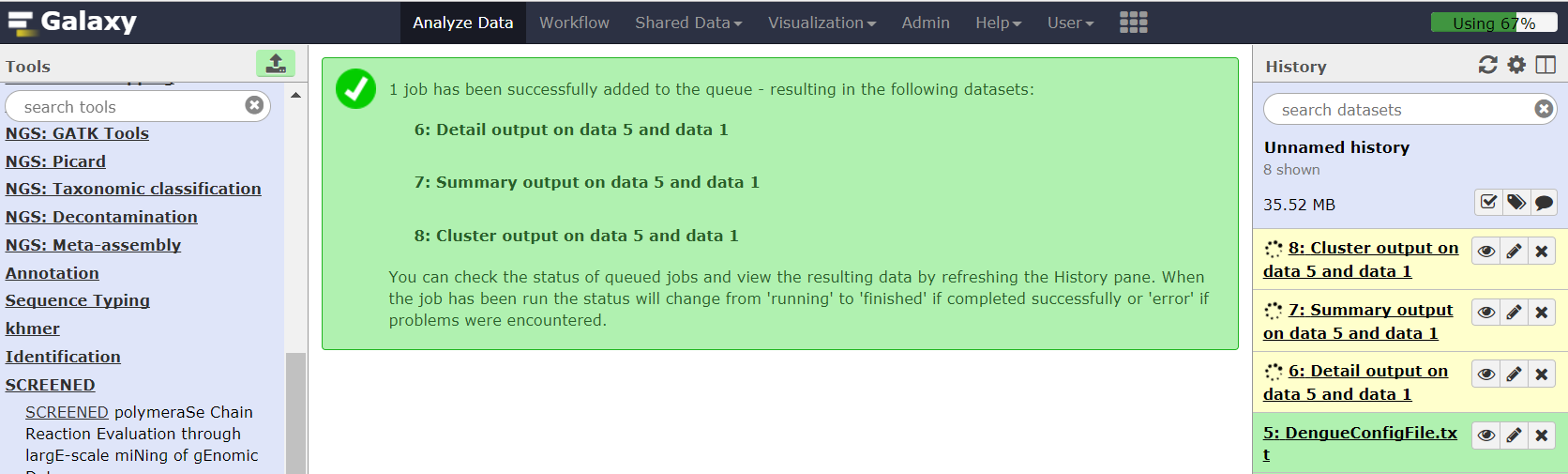


**Figure SI7:** Running SCREENED. The green box in the middle panel indicates the tool has been launched, and the yellow boxes in the History panel indicate the tool is running.

### Viewing and downloading output

Three tab-delimited output files will be generated once SCREENED is done analysing the input data. The detailed output file contains extensive information for every analysed genome regarding the tested selection criteria. The summary output file contains a concise overview of which genomes can be successfully detected *in silico*. Lastly, the cluster output file will contain the clusters of individual targeted genomic regions in all analysed genomes if this option was set (see below). Click on the box ‘6: Detail output on data 5 and data 1’ to expand this box. Pressing the ‘eye icon’ will show the output in the middle panel. Pressing the ‘download icon’ will allow to download the file. The first lines starting with ‘#’ are comments providing information on which options were used when running SCREENED. The last line starting with ‘#’ contains the column headers for the analysed genomes in the rows underneath (see Figure SI8). Other output files can be viewed or downloaded similarly.


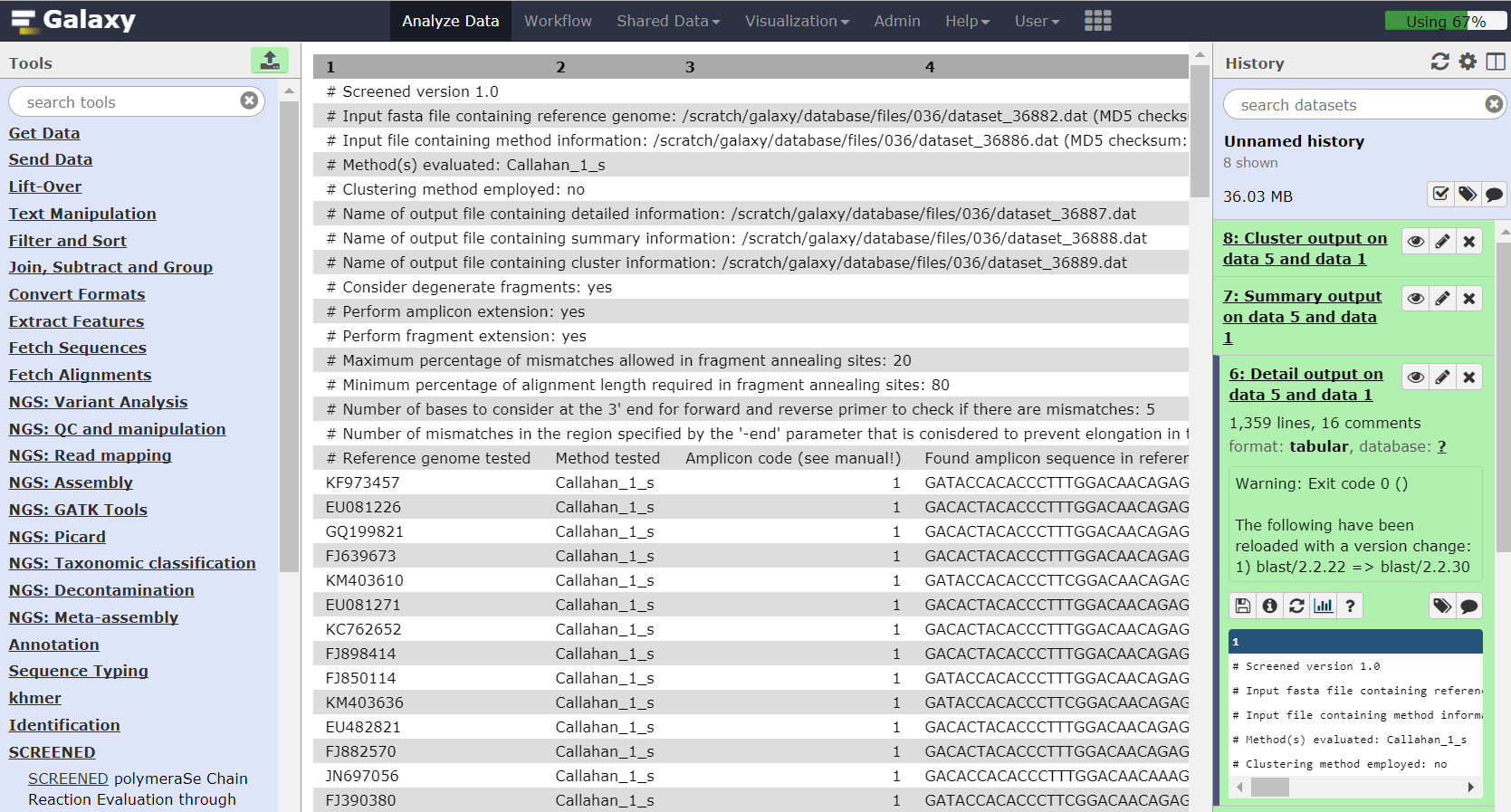


**Figure SI8:** Accessing and downloading SCREENED output. The ‘eye icon’ allows to see the results of the analysis within Galaxy. The ‘download icon’ allows to download the results to your local computer.

### Analysing output

The file ‘7: Summary output on data 5 and data 1’ will contain a concise overview of results for the method, by listing all genomes that were detected. The file ‘6: Detail output on data 5 and data 1’ will contain a detailed overview of results that will enable to reconstruct information such as presented in Table 2 of the main manuscript. Note that this file can be opened in Excel for more extensive manipulations.

### Additional options and analyses

SCREENED allows for additional advanced options to be set for more in-depth analysis that can be accessed by selecting ‘Yes’ for ‘Set advanced options?’ (see Figure SI9).


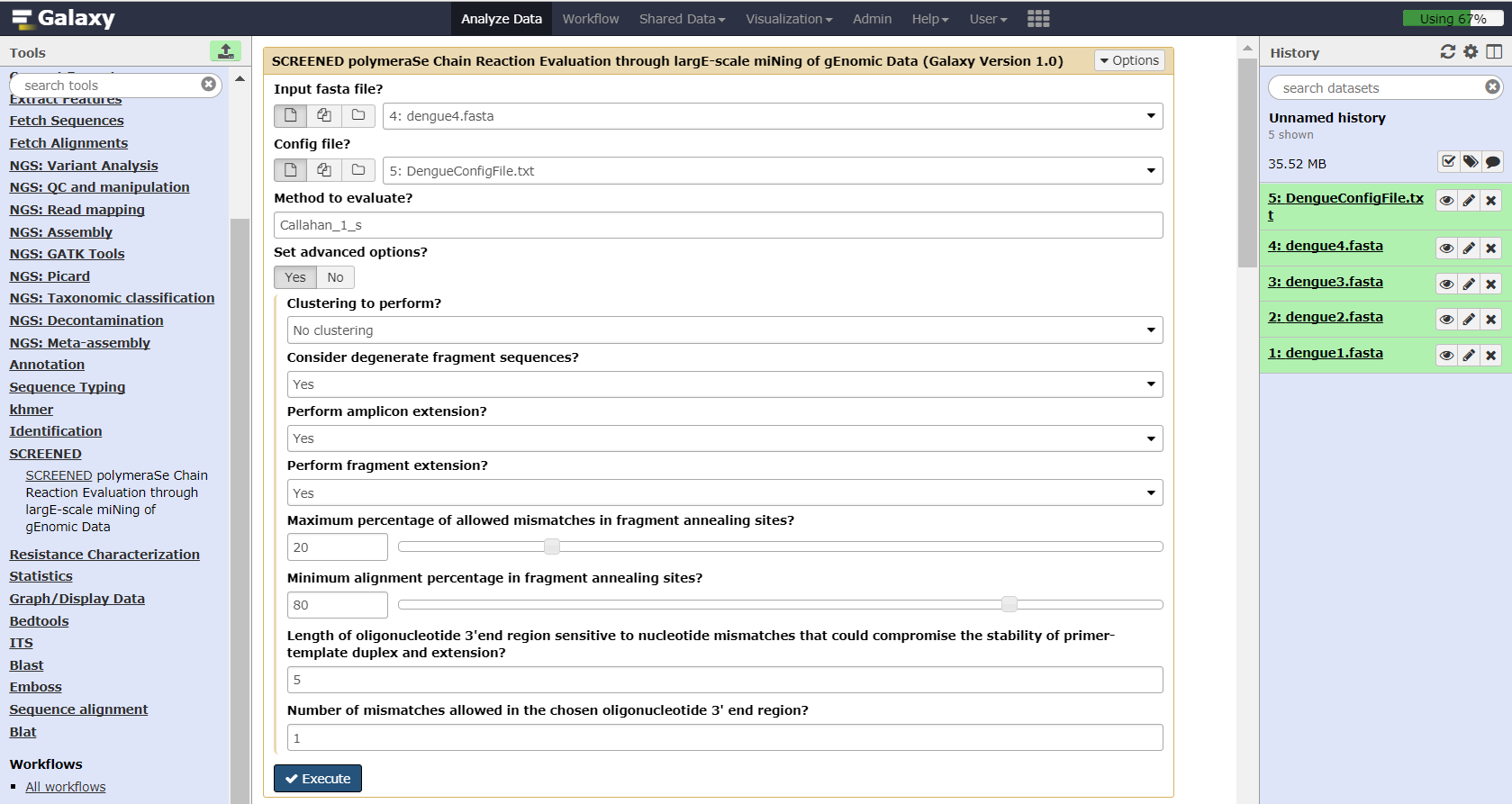


**Figure SI9**: Advanced options of SCREENED.

It is possible to also perform an additional clustering step that will group all recovered targeted genomic regions based on sequence similarity for investigative purposes. Evaluating all degenerate fragment possibilities can also be turned off (not recommended). Automated amplicon and fragment extension can also be turned off (not recommended). Of particular interest for most users are the four last options, which allow to change the default threshold values for employed selection criteria. There is a detailed help section underneath the tool interface in the middle panel that provides much more information for all these advanced options.

When analysing other methods and genomes, ensure that the input fasta and config files are properly formatted to ensure SCREENED can run. For fasta files, every genome should be represented by a unique header followed on the next line by the genome sequence that can only contain IUPAC nucleotide characters, adhering to the following format:

>genome1

AAAAAAAAAAAAAAAAAAAAAAAAAAAAAAAAAAAAAAA

>genome2

CCCCCCCCCCCCCCCCCCCCCCCCCCCCCCCCCCCC

(etc.)

Note that only whole genomes are supported, i.e. the entire genome needs to be present and cannot be broken over different contigs. Genomes consisting out of multiple contigs can however still be analysed by concatenating all contigs in a single line head to tail for analysis. Ensure that the type of new fasta files is set to ‘fasta’ when uploading (see Figure SI5), or they will not be recognized by Galaxy as being fasta files.

The config file should be created in tab-delimited format where each line represents all information for one RT-qPCR method. Information for multiple RT-qPCR methods can be in the same config file, as long as each method is added as an extra line. The first column should always contain a unique method identifier (avoid complex characters such as ‘&’, ‘*’ etc.). The second, third, fourth, and fifth columns should contain the sequence information for the forward primer, reverse primer, probe, and template reference for the method. All sequence information should be present in a 5’ -> 3’ direction, and only contain IUPAC characters. In case no template reference is known, this should be created manually by using tools such as BLAST [5] to find the location of the forward and reverse primers on a reference genome of interest, by which the template reference sequence to search for this specific targeted region in other genomes will be defined (i.e. the sequence on the reference genome between the forward and reverse primers, including the primer regions). Ensure that the type of the new config file is set to ‘tabular’ when uploading (see Figure SI5), or it will not be recognized by Galaxy as being a config file.

The specific RT-qPCR method can then be analysed by providing its name under ‘Methods to evaluate?’ (see Figure SI6). Note that the name of the method typed in this field should correspond to one of the RT-qPCR methods for which information is present in the config file, and is case-sensitive. There is also a detailed help section underneath the tool interface in the middle panel that provides more information for running the analysis on different datasets. This help section will always contain the most up-to-date information for running SCREENED.

## References

1. Leparc-Goffart I, Baragatti M, Temmam S, Tuiskunen A, Moureau G, Charrel R+ et al. Development and validation of real-time one-step reverse transcription-PCR for the detection and typing of dengue viruses. Journal of Clinical Virology. 2009; 45: 61-66.

2. Chien LJ, Liao TL, Shu PY, Huang JH, Gubler DJ, Chang GJ. Development of Real-Time Reverse Transcriptase PCR Assays To Detect and Serotype Dengue Viruses. J Clin Microbiol. 2006; 44: 1295-1304.

3. Afgan E, Baker D, van den Beek M, Blankenberg D, Bouvier D, Cech M et al. The Galaxy platform for accessible, reproducible and collaborative biomedical analyses: 2016 update. Nucleic Acids Res. 2016; 44: W3-W10.

4. Callahan JD, Wu SJ, Dion-Schultz A, Mangold BE, Peruski LF, Watts DM et al. Development and Evaluation of Serotype- and Group-Specific Fluorogenic Reverse Transcriptase PCR (TaqMan) Assays for Dengue Virus. J Clin Microbiol. 2001; 39: 4119-4124.

5. Camacho C, Coulouris G, Avagyan V, Ma N, Papadopoulos J, Bealer K et al. BLAST+: architecture and applications. BMC Bioinformatics. 2009; 10: 421.
